# Spatially Localized Entropy-Driven Evolution of Nucleic Acid-Based Constitutional Dynamic Networks for Intracellular Imaging and Spatiotemporal Programmable Gene Therapy

**DOI:** 10.1021/jacs.4c03651

**Published:** 2024-07-16

**Authors:** Nina Lin, Yu Ouyang, Yunlong Qin, Ola Karmi, Yang Sung Sohn, Songqin Liu, Rachel Nechushtai, Yuanjian Zhang, Itamar Willner, Zhixin Zhou

**Affiliations:** † School of Chemistry and Chemical Engineering, Southeast University, Nanjing 211189, China; ‡ Institute of Chemistry, The Hebrew University of Jerusalem, Jerusalem 91904, Israel; § Institute of Life Science, The Hebrew University of Jerusalem, Jerusalem 91904, Israel

## Abstract

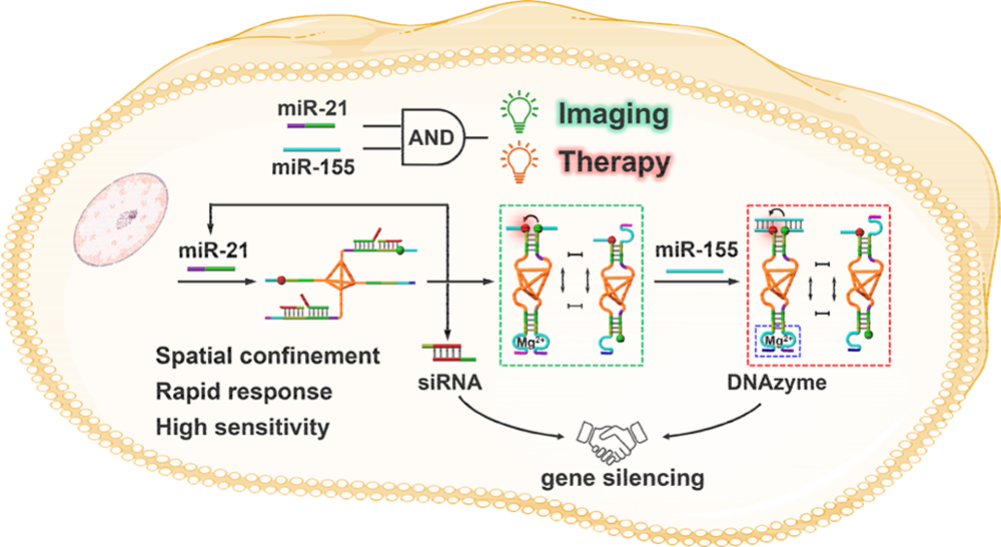

The primer-guided entropy-driven high-throughput evolution of the DNA-based constitutional dynamic network, CDN, is introduced. The entropy gain associated with the process provides a catalytic principle for the amplified emergence of the CDN. The concept is applied to develop a programmable, spatially localized DNA circuit for effective *in vitro* and *in vivo* theranostic, gene-regulated treatment of cancer cells. The localized circuit consists of a DNA tetrahedron core modified at its corners with four tethers that include encoded base sequences exhibiting the capacity to emerge and assemble into a [2 × 2] CDN. Two of the tethers are caged by a pair of siRNA subunits, blocking the circuit into a mute, dynamically inactive configuration. In the presence of miRNA-21 as primer, the siRNA subunits are displaced, resulting in amplified release of the siRNAs silencing the HIF-1α mRNA and fast dynamic reconfiguration of the tethers into a CDN. The resulting CDN is, however, engineered to be dynamically reconfigured by miRNA-155 into an equilibrated mixture enriched with a DNAzyme component, catalyzing the cleavage of EGR-1 mRNA. The DNA tetrahedron nanostructure stimulates enhanced permeation into cancer cells. The miRNA-triggered entropy-driven reconfiguration of the spatially localized circuit leads to the programmable, cooperative bis-gene-silencing of HIF-1α and EGR-1 mRNAs, resulting in the effective and selective apoptosis of breast cancer cells and effective inhibition of tumors in tumor bearing mice.

## Introduction

The structural and functional information encoded in the base sequence of nucleic acids provides the foundation for the development of the area of dynamic DNA nanotechnology.^1−3^ Diverse functional systems including switches,^4^ machines,^5^ dynamically self-assembled nanostructures,^6−8^ stimuli-responsive DNA materials,^9−11^ and logic circuits^12,13^ were assembled based on these properties of the nucleic acids. Different applications of these structures were reported for sensing,^14−17^ controlled drug delivery,^18,19^ guided assembly of programmed nanoparticle structures,^20−23^ and the construction of nanoscale optical and electronic devices.^24,25^ In addition, the high programmability of nucleic acids dictated toehold-mediated strand displacement reactions,^26,27^ entropy-driven DNA reactions,^28,29^ catalytic hairpin assembly reactions,^30^ hybridization chain reactions,^31,32^ and exponential chain reactions using DNAzyme/ribozyme.^33,34^ Particularly, entropy-driven DNA reactions were broadly applied to develop computing circuits,^35,36^ molecular engineering,^37,38^ nanostructure self-assembly,^39^ enzyme activity modulation,^40^ and biosensing.^29,41,42^

The programmed hybridization and strand displacement reactions were extensively used to construct dynamically reconfigurable DNA networks^43,44^ and to assemble transient dissipative networks.^45,46^ Constitutional dynamic networks (CDNs) revealing adaptive,^47^ hierarchically adaptive,^48^ feedback-driven,^49^ and intercommunication^50^ features were reported. Diverse applications of the dynamic networks for network-guided operation of biocatalytic cascades,^51^ orthogonal and transient biocatalytic cascades,^52^ and switchable material functions^53^ were demonstrated. While significant advances in the construction of DNA networks and the uses of the systems were demonstrated, important challenges are still ahead of us. The effective assembly and delivery of DNA reaction networks into the cells, retaining the functional intracellular integrity of the networks, and the capacities to probe and image their intracellular functionalities are still scientific challenges. DNA nanostructures, such as DNA tetrahedra, proved to be effective delivery vehicles of payloads, and stimuli-responsive loaded carriers were employed as drug delivery agents.^54,55^ The use of functional cell-permeating carriers for the intracellular triggered evolution of networks by a catalytic DNA circuit could provide an effective means to yield an intact reaction network in cells. Indeed, several reports recently addressed the evolution of CDNs,^56−58^ and these concepts could be adapted for intracellular assembly of dynamic networks. Besides the precise delivery of DNA networks into cells, emerging functions, particularly related to programmed gene regulation and potential gene therapy, by the networks could be envisaged.

In this study, we introduce the primer-induced entropy-driven evolution of CDNs, revealing inherent amplification features toward the dynamic emergence of the CDN. By coupling two reaction circuits, the primer-induced, entropy-driven cascaded evolution of two CDNs is demonstrated. The concept of primer-induced entropy-driven evolution is then applied to assemble a localized DNA tetrahedron core, functionalized at its corners with “caged” oligonucleotide tethers as a stimuli-responsive framework. The localized DNA circuits on the tetrahedron undergo, in the presence of an auxiliary primer, the entropy-driven, strand-displacement-guided dynamic reconfiguration of the “caged” framework into a CDN, composed of two tetrahedral core units to which four dynamic constituents are conjugated. The dynamic reconfiguration of the reaction circuit to the CDN involves the release of the caging strands and reveals primer-induced amplification and fast emergence kinetics features, dictated by the core tetrahedron confined reaction module. By substitution of the blocker units associated with the localized circuit with a pair of RNAs comprising a siRNA duplex (s/as), reprogramming of the primer strand with a target miRNA-21, and appropriate engineering of the DNA tethers linked to the tetrahedron core of the DNA circuit, the miRNA-21-induced reorganization of the circuit into the dynamically equilibrated CDN proceeds while releasing the siRNA duplex from the localized circuit. The released siRNA duplex is pre-engineered to silence HIF-1α mRNA involved in cell apoptosis. The resulting evolved CDN is, however, predesigned to include a DNAzyme acting as a catalyst cleaving the mRNA expressing EGR-1 involved in cell apoptosis, too. Furthermore, the evolved CDN is dynamically reconfigured by a second miRNA (miRNA-155), thereby upregulating the EGR-1 mRNA cleavage activity of the DNAzyme. That is, the dynamic operation of the miRNA-responsive localized circuit leads to the cooperative gene silencing of the mRNAs expressing EGR-1 and HIF-1α, two key proteins operating in the cancer cell viability cycle. Making use of the enhanced permeability of DNA tetrahedron structures into cancer cells, the miRNA-responsive DNA tetrahedra reaction module was applied as a functional framework operating the entropy-driven reaction circuit, leading to the amplified release of the siRNA duplexes silencing the HIF-1α mRNA and to the evolution of the miRNA-155-responsive CDN silencing the mRNA expressing EGR-1. *In vitro* cell experiments, subjecting MCF-7 or MDA-MB-231 breast cancer cells and normal control LX-2 cells to the bis-gene silencing reaction circuit, demonstrated effective and selective apoptosis of the cancer cells. *In vivo* experiments further supported the effective inhibition of MDA-MB-231 tumor growth by the gene-regulating reaction module in xenograft tumor bearing mice.

## Results and Discussion

**Figure 1:**
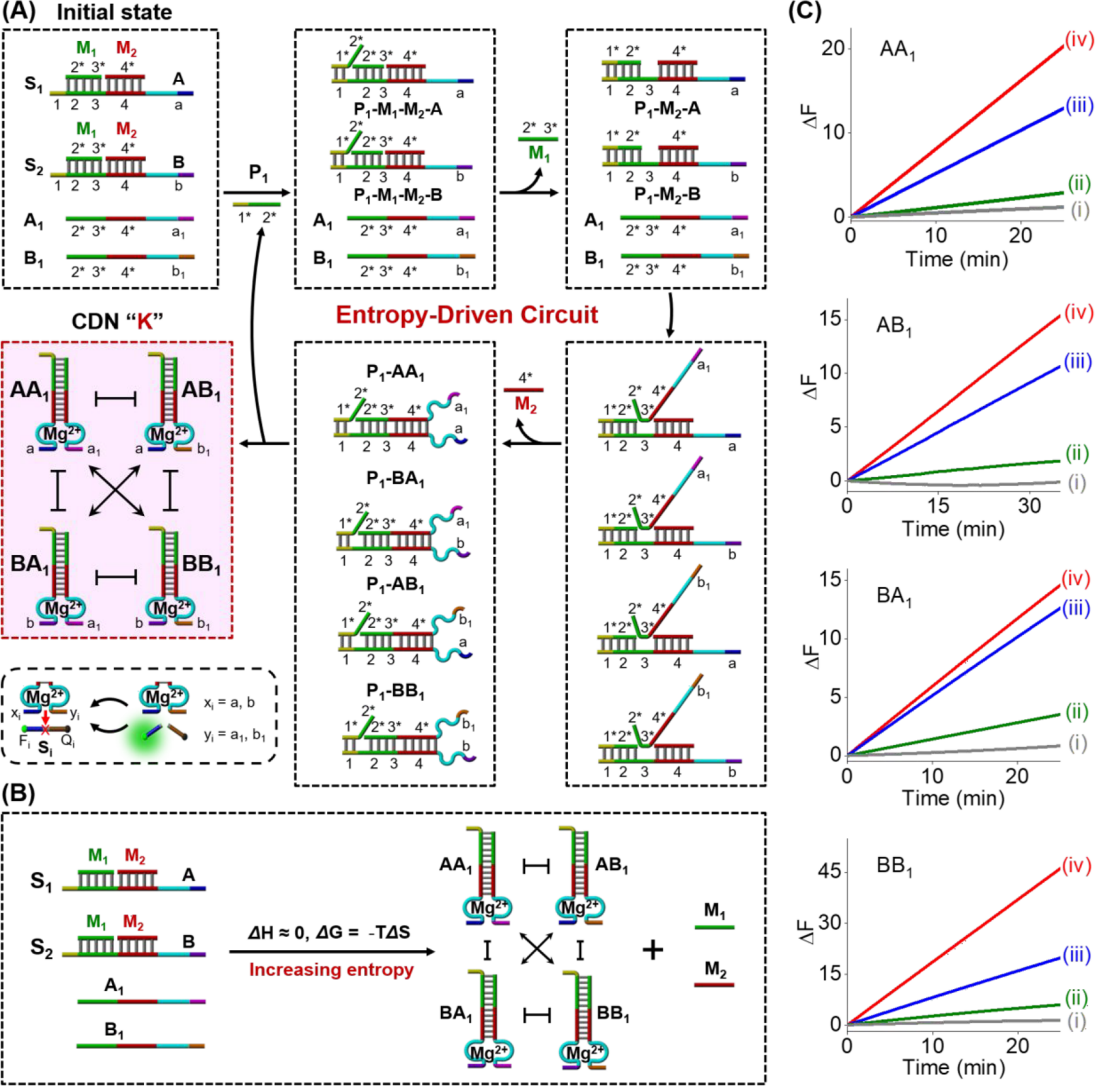
(A) Schematic reaction module of P_1_-triggered nonenzymatic entropy-driven catalytic DNA circuit leading to the evolution of a [2 × 2] CDN “K”. (B) Schematic thermodynamic parameters associated with the P_1_-triggered entropy-driven transition of the reaction module into CDN “K” and M_1_/M_2_ products. (C) Time-dependent fluorescence changes generated upon the cleavage of the substrates by the DNAzyme reporter units associated with the constituents of CDN “K” evolved in the presence of variable concentrations of P_1_: (i) 0 nM, (ii) 1 nM, (iii) 10 nM, and (iv) 1 μM.

The principle to design the catalyzed evolution of a [2 × 2] CDN “K” using nonlocalized entropy-driven DNA circuits is outlined in Figure 1A. The parent reaction module includes two three-stranded substrate complexes, A-M_1_-M_2_ (S_1_) and B-M_1_-M_2_ (S_2_), and two fuel strands, A_1_ and B_1_. The primer P_1_ first hybridizes with the substrates S_1_ and S_2_ through toehold 1-mediated strand displacement, leading to the release of metabolite strand M_1_ to generate metastable three-stranded DNA structures, P_1_-M_2_-A and P_1_-M_2_-B. These metastable structures are exposed to single-stranded toehold domain 3 facilitating the binding of fuel strands A_1_ and B_1_ that simultaneously displace the two P_1_ strands and metabolite M_2_ strands. The process results in the recycling of primer P_1_ and the amplified generation of four duplex constituents, AA_1_, AB_1_, BA_1_, and BB_1_, comprising the dynamically equilibrated CDN “K”. The net dynamic transition is shown in Figure 1B. The total number of base pairs between the reactants and products remains unchanged, leading to Δ*H* ≈ 0. Thus, the process is thermodynamically driven forward by the entropy gain of the released DNA molecules M_1_ and M_2_. According to the van’t Hoff equation and using NUPACK software, the reaction efficiency of the entropy-driven DNA circuit is estimated to be more than 99%. (For evaluation of the entropy, the free energy values associated with the entropy-driven evolved CDN, and the estimated efficiency yield of the constituents, see page S9 and accompanying discussion). Compared with kinetically controlled hairpin-based systems driven by the energy of base-pair formation,^57^ the entropy-driven DNA circuit presented here reveals significantly higher signal gain, modularity, and simplicity, which is essential for scaling up to larger circuits.^16,28^

Each of the resulting constituents in CDN “K” includes a Mg^2+^-dependent DNAzyme unit that differs in the single-stranded arms for the selective cleavage of the fluorophore/quencher (F_i_/Q_i_)-functionalized substrates, S_i_. The DNAzyme reporter units provide reliable readout signals to quantitatively assess the concentrations of the constituents in CDN “K”. Figure 1C shows the time-dependent fluorescence changes generated by the four DNAzyme reporter units associated with the CDN “K” evolved by the entropy-driven circuit at variable concentrations of P_1_ shown in Figure 1A. By following the rates of cleavage of the F_i_/Q_i_-functionalized substrates and using appropriate calibration curves correlating the cleavage rates of the respective substrates to the concentrations of the intact constituents, Figures S1 and S2, the concentrations of the constituents in the dynamically equilibrated CDN “K” were evaluated, and their concentrations are summarized in Table 1. Evidently, in the presence of 1 nM primer P_1_, the nonenzymatic amplified entropy-driven DNA circuit leads to the evolved CDN “K” composed of the components A, A_1_, B, and B_1_ in the concentration range of ca. 0.16 μM. That is, an amplified process corresponding to a ca. 160-fold increase in the contents of the equilibrated CDN constituents, as compared to the fuel strand P_1_, is observed, demonstrating the significantly high signal gain and catalytic efficiency of the entropy-driven circuit. Figure S3 depicts the quantitative polyacrylamide gel electrophoresis evaluation of the emergence of the CDN “K” by the entropy-driven DNA circuits. The contents of the constituents obtained by the quantitative electrophoretic experiments are summarized in Table S1, and the resulting constituent contents are consistent with those determined by the DNAzyme reporter units.

**Table 1:** Concentrations of Constituents Associated with CDN “K” Evolved by the P_1_-Stimulated Activation of the Entropy-Driven DNA Circuit Shown in Figure 1A

P_1_	CDN	AA_1_	AB_1_	BA_1_	BB_1_
1 μM	K^a^	0.40	0.37	0.37	0.48
10 nM	K^a^	0.23	0.23	0.25	0.21
1 nM	K^a^	0.08	0.09	0.08	0.07

a The concentrations of the constituents (μM) were evaluated from the time-dependent fluorescence changes generated by the DNAzyme reporter units and using appropriate calibration curves in Figures S1 and S2.

**Figure 2:**
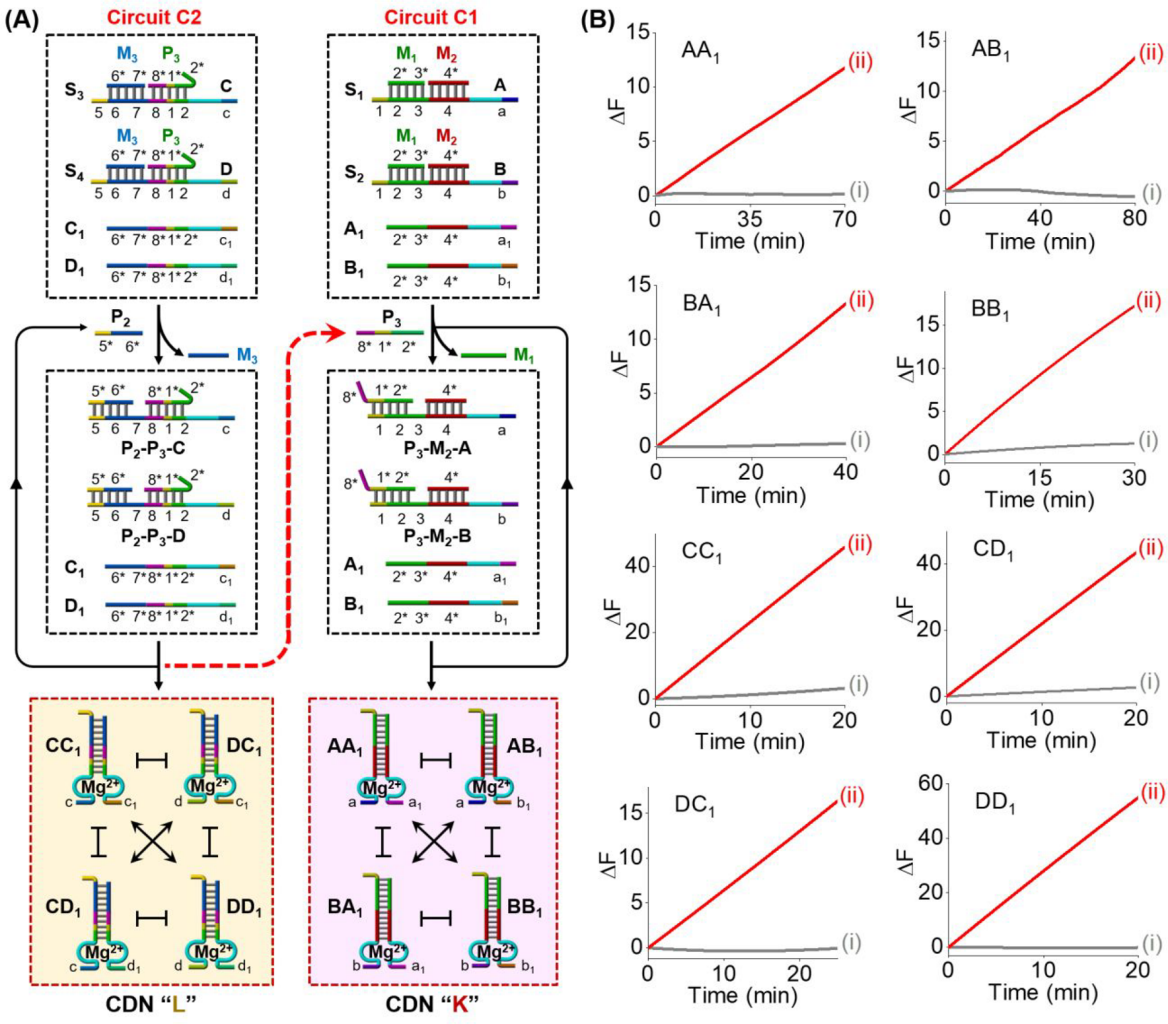
(A) Schematic two-layer cascaded entropy-driven DNA circuits, C2 and C1, leading to the evolution of CDN “L” and CDN “K”: the primer P_2_ initiates subcircuit C2 for the entropy-driven evolution of CDN “L”, and the concomitant release of primer P_3_ activates the cascaded subcircuit C1 to yield CDN “K”. (B) Time-dependent fluorescence changes generated by the DNAzyme units associated with the constituents in CDN “L” and CDN “K” upon the activation of the two-layer entropy-driven cascaded process: (i) in the absence of primer P_2_ and (ii) in the presence of P_2_.

To demonstrate the ability of modular circuit design and scalability using entropy-driven DNA circuit, we designed a two-layer cascaded emergence of CDNs using cascaded entropy-driven DNA circuits. This is exemplified in Figure 2A by introducing an upstream subcircuit C2 whose output serves as the catalyst for downstream subcircuit C1. The introduction of primer P_2_ activates the upstream subcircuit C2, leading to the continuous assembly of CDN “L” consisting of CC_1_, CD_1_, DC_1_, and DD_1_. The P_3_ strand that was caged inside the parent three-stranded complexes P_3_-M_3_-C and P_3_-M_3_-D to prohibit the direct activation of the downstream subcircuit C1 was released from the upstream circuit C2. The free strand P_3_ triggered the downstream subcircuit C1, thus resulting in the amplified generation of the duplex constituents AA_1_, AB_1_, BA_1_, and BB_1_ comprising CDN “K”. Each of constituents in evolved CDNs “L” and “K” includes DNAzyme reporter units that quantitatively evaluate the contents of the constituents. Figure 2B depicts the time-dependent fluorescence changes generated by the eight DNAzyme reporter units in the evolved CDNs “L” and “K. All eight DNAzyme reporter units were activated upon the triggering of circuits C2 and C1 with P_2_, Figure 2B, indicating the cascaded emergence of two equilibrated CDNs “L” and “K”. A control experiment, Figure S4, indicated that the primer P_2_ could not activate the separate downstream subcircuit C1, confirming that the cascaded evolution of CDNs originates, indeed, from the intercommunication of the two-layer entropy-driven DNA circuits. Using the respective calibration curves in Figures S1 and S2, the concentrations of the constituents in the resulting evolved CDNs “K” and “L” were evaluated, and these are summarized in Table S2. Gel electrophoresis experiments further confirmed the cascaded emergence of CDNs “K” and “L”, Figure S5. The contents of the constituents were quantitatively analyzed by the respective gel electrophoretic bands, and their contents are summarized in Table S2. The values are in good agreement with the concentration values determined by the DNAzyme reporter units.

The results presented in Figure 2 demonstrate the feasibility to operate a cascaded entropy-driven DNA circuit, revealing modularity and amplifying capacity for the enzyme-free catalyzed emergence of CDNs with high signal gain. An obvious disadvantage of the entropy-driven reaction module generating the CDNs is, however, the slow emergence of the equilibrated networks, originating from the free diffusion of the reactants and intermediate products at realistic low concentrations.^59,60^ To overcome these difficulties, the operation of the entropy-driven DNA circuit should be activated in a confined reaction volume or a confined supramolecular framework.^61^ Indeed, recent reports demonstrated the advantages of confined microenvironments or structurally engineered scaffolds as reaction media for enhanced biochemical reactions, as compared to diffusional systems.^62^ Microdroplets,^63,64^ liposomes,^65^ particles,^15,66^ and microcapsules^67,68^ were reported as confined reaction volumes to enhance catalytic reactions; supramolecular frameworks, such as DNA strips^69^ and origami^70,71^ were used to spatially organize biomolecules for enhanced cascaded catalysis; and cell membranes acted as fluidly confined systems to improve reaction efficiency.^72^ With the vision that we aim to apply the entropy-driven DNA circuits as functional reaction modules for therapeutic sense-and-treat applications, we searched for supramolecular frameworks to operate the circuits that fit these goals. The DNA tetrahedron demonstrated effective cell permeation efficacies and significantly enhanced biostability against nuclease degradation in a biological environment, as compared to duplex nucleic acid structures.^73^ Moreover, the functionalization of the corners of DNA tetrahedra with recognition molecules, e.g., aptamers^74^ or proteins,^75^ allowed the targeting of the DNA tetrahedra to specific cells and their applications in diagnostics and therapeutics.^76,77^

**Figure 3:**
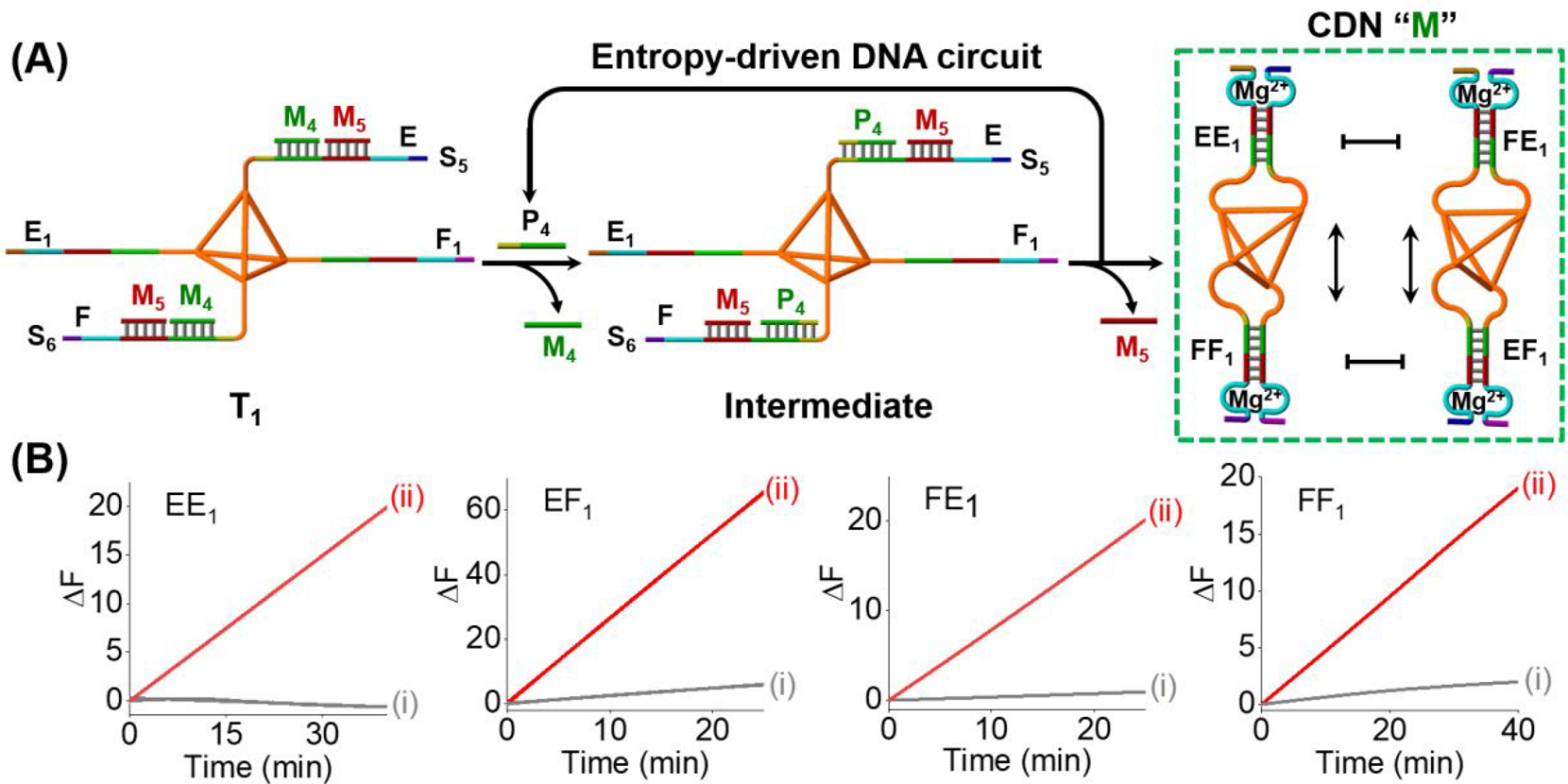
(A) Schematic of spatially localized DNA circuit consisting of a DNA tetrahedron functionalized at its corners with DNA tethers that allows the P_4_-activated entropy-driven DNA circuit to lead to the dynamic assembly of CDN “M”. The equilibrated CDN “M” includes four interequilibrated constituents, EE_1_, FF_1_, FE_1_, and EF_1_, where constituents, EE_1_ and FF_1_ are anchored to one DNA tetrahedron core, and constituents FE_1_ and EF_1_ are functionalized on a second DNA tetrahedron core. (B) Time-dependent fluorescence changes generated by the DNAzyme reporter units: (i) in the absence of P_4_; (ii) upon subjecting the P_4_ to the localized circuit.

Accordingly, we applied the inert DNA tetrahedra as functional carriers to operate the entropy-driven DNA circuit. Figure 3 exemplifies the spatially localized entropy-driven DNA circuit for the fast emergence of a CDN. The DNA tetrahedra were modified at their corners with two substrate strands, S_5_ and S_6_, and two fuel strands, E_1_ and F_1_. The substrate strands, S_5_ and S_6_, were engineered to include sequence domains, E and F complementary to E_1_ and F_1_, yet these domains were partially protected with M_4_ and M_5_ strands, and thus interhybridization of the fuels/substrates was prohibited (Figure S6). Each assembly step associated with the tetrahedron structure was characterized by gel electrophoresis, Figure S7 and accompanying discussion. The introduction of primer P_4_ could activate the spatially localized entropy-driven DNA circuits to generate a dynamically equilibrated CDN “M”. The primer P_4_ displaces the strands M_4_ from S_5_ and S_6_, generating toehold domains between M_5_ and P_4_. The newly exposed toehold domains then allow the hybridization of E_1_ or F_1_, resulting in the displacement of M_5_ and P_4_ and the formation of two tetrahedra, each functionalized with two constituents EE_1_/FF_1_ or FE_1_/EF_1_. While the primer P_4_ continuously initiates the parent DNA circuit, the two released DNA strands, M_4_/M_5_, accumulate over time as waste products, and the dynamic interexchange between the resulting constituent-modified tetrahedra leads to the formation of equilibrated CDN “M”. The substrate strands, S_5_ and S_6_, and fuel strands, E_1_ and F_1_, are pre-engineered, however, to yield constituents functionalized each with a different DNAzyme unit, providing a catalytic transducer for quantitative evaluation of the contents of the constituents. Figure 3B shows the time-dependent fluorescence changes generated by DNAzyme reporter units associated with the equilibrated CDN “M”. By using the appropriate calibration curves displayed in Figures S8 and S9, the contents of the constituents in the equilibrated CDN “M” were quantitatively evaluated, and the corresponding contents of the constituents are summarized in Table S3. Assessment of the contents of the constituents was further supported by quantitative gel electrophoresis experiments, and the results are presented in Figure S10. It should be noted that the primer-induced entropy-driven localized reconfiguration of the DNA-tetrahedral circuit T_1_ to CDN “M” reveals a low leakage phenomenon, often associated with strand displacement processes, resulting in a high signal-to-noise readout signal. This originates from optimized pre-engineering of the reaction circuits (for detailed experimental results and discussion, see Figure S6).

The benefits of the DNA tetrahedron as a functional carrier for operating an entropy-driven evolution circuit are reflected by fast assembly of the circuit originating from the spatially confined interaction of the circuit components, the amplification features of the circuit, the enhanced cell-permeation efficacy, and improved biostability against biodegradation. Beyond these advantages, the spatially localized circuit could introduce, by appropriate structural engineering, important functionalities, particularly related toward programmed gene regulation and potential gene therapy: (i) The self-assembly of the localized CDNs yields constituents conjugated to DNAzymes as reporter units. One may, however, design a DNAzyme sequence that could cleave a target mRNA thereby leading to the regulation of gene expression patterns in cells. (ii) The dynamic reconfiguration of the localized CDN can be triggered by further input. The upregulation of the DNAzyme-linked constituent cleaving the mRNA could further regulate the gene expression level. (iii) In the canonical entropy-driven evolution systems, the released metabolite strands, M_1_–M_6_, accumulate over time and turn into waste products after being used only once, which limits the catalytic efficiency and functional benefits of DNA circuits. The waste products associated with the localized circuit (e.g., M_4_ and M_5_ in the model system, Figure 3) can be exchanged with appropriately engineered functional RNA strands, e.g., small interfering RNAs (siRNAs).^78^ That is, upon the triggered entropy-driven formation of the CDNs, predesigned siRNA duplexes are released that may, then, activate RNA interference (RNAi) catalyzing the cleavage of a further and different mRNA participating in the gene expression process. (iv) The localized circuit might be initiated, however, by endogenous microRNA (miRNA/miR). This is important to induce selectivity of the localized circuit for the precisely targeted delivery of functional nucleic acids to specific cells/organs. miRNAs are important endogenous oncogene biomarkers, and thus, their use as initiators of the localized circuit is anticipated to lead to precise therapeutic treatment toward cancer cells.^79^ While the concentrations of endogenous miRNAs are usually low, the recycling of the miRNA primers by the localized circuit could provide an amplification path for intracellular operation of the circuit. That is, the spatially localized circuit may be coupled to multiple miRNA triggers and functional nucleic acids (e.g., siRNAs and mRNA-cleaving DNAzymes) that operate synergistic input-guided gene regulation pathways with potential targeted therapy. With this vision, we sought to design an entropy-driven circuit localized on DNA tetrahedra driven by multiple miRNA triggers and capable of synergistic control of gene expression pathways through the generation of functional mRNA-cleaving DNAzymes and siRNAs. This model system will then be adapted for spatial and selective control of gene expression in diverse cellular environments. It should be noted that, to the best of our knowledge, the use of entropy-driven DNA circuits in cellular environments for potential therapeutic applications is unprecedented.

**Figure 4:**
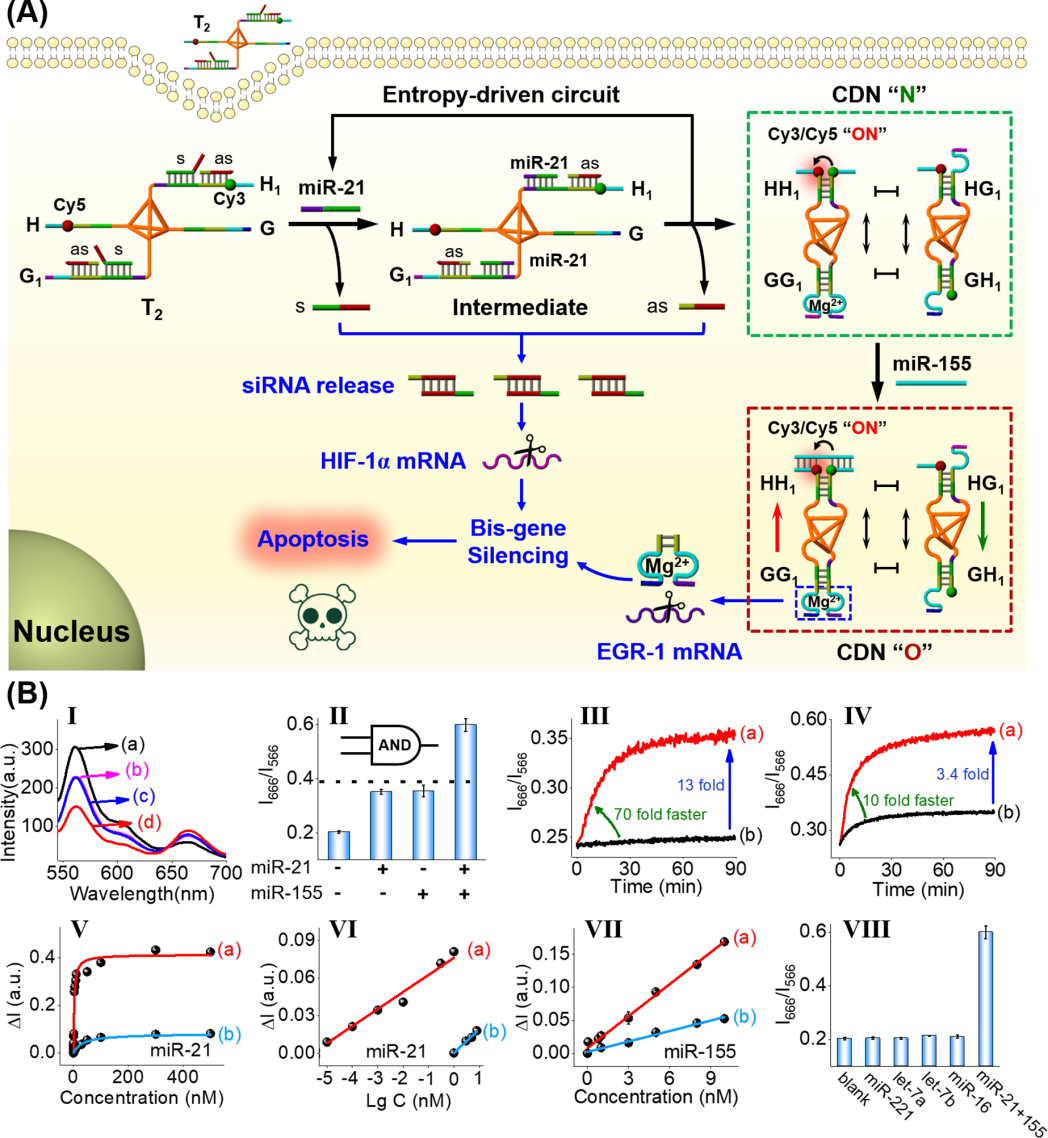
(A) Schematic application of spatially localized DNA circuit activated by miRNA primers that leads to the cooperative gene silencing of HIF-1α mRNA and EGR-1 mRNA causing cancer cell apoptosis. (B) Panel I, FRET responses of localized circuit: (a) in the absence of miR-21 and miR-155, (b) in the presence of miR-21 leading to the formation of CDN “N”, (c) in the presence of miR-155, and (d) in the presence of miR-21 and miR-155. Panel II, FRET intensities transduced by the localized circuit, under different conditions, expressed as *I*_666_(Cy5)/*I*_566_(Cy3) demonstrating the highest FRET following “AND” logic gate. Panel III: (a) Temporal FRET intensities generated, upon the miR-21-activated entropy-driven transformation of the localized circuit into CDN “N”. (b) Temporal FRET intensities of the miRNA-21-activated nonlocalized circuit consisting of diffusional, separated components lacking the tetrahedral core units. Panel IV, Temporal FRET intensity changes upon (a) subjecting the localized CDN “N” to miR-155 leading to the formation of CDN “O” and (b) subjecting the diffusional mixture of CDN “N” without the core tetrahedra units, to miR-155. Panel V, FRET intensities generated upon subjecting the circuits to variable concentrations of miR-21: (a) spatially localized circuit; (b) nonlocalized circuit. Panel VI, Calibration curves relating FRET intensities to the logarithm of the miR-21 concentrations in (a) spatially localized circuit and (b) nonlocalized circuit. Panel VII, Calibration curve corresponding to the FRET intensity change in the presence of variable concentrations of miR-155: (a) spatially localized circuit; (b) nonlocalized circuit. Panel VIII, FRET intensities generated by spatially localized circuit subjected to miR-21 and miR-155 in comparison to FRET intensities transduced by localized circuit subjected to a set of foreign miRs. The results demonstrate the selective response of the spatially localized circuit to miR-21 and miR-155.

The principle of the miRNA-responsive intelligent theranostic platform using a spatially localized DNA circuit is exemplified in Figure 4. The spatially localized circuit consists of a tetrahedron T_2_ modified at its corners with four DNA tethers, H, G, H_1_, and G_1_. The tethers H_1_ and G_1_ are blocked by RNA strands (s) and (as), acting as sense and antisense RNAs capable of assembling into duplexed siRNAs. The hybridization of the complementary s/as strands with the tethers G_1_ and H_1_ prohibits, however, any RNAi activity. In addition, strands H and H_1_ are internally modified with the fluorophores Cy5 and Cy3. Subjecting the localized circuit to miR-21 displaces the blocker unit (s), leading to the generation of toehold domains on tethers H_1_ and G_1_. The newly exposed toehold domains allow the interhybridization of tethers H and G followed by the displacement of the miR-21 and blocker units (as), thus yielding two dynamically equilibrated tetrahedra comprising CDN “N”, where one tetrahedron includes the two constituents HH_1_/GG_1_ and the second tetrahedron is functionalized with the constituents HG_1_/GH_1_. The formation of CDN “N” involves, however, the regeneration of the miR-21, and thus an amplification path for the miR-21 input. Also, the continuous release of strand “s” and strand “as” spontaneously assembles into the active siRNA duplexes to efficiently activate the RNAi therapy. The constituents in CDN “N” are pre-engineered to respond to a second trigger, miR-155. Subjecting CDN “N” to miR-155 results in the reconfiguration of CDN “N” to CDN “O”, where the constituent HH_1_ is stabilized. This results in the upregulation of HH_1_, the concomitant upregulation of the constituent GG_1_, and the downregulation of HG_1_ and GH_1_. The constituent GG_1_ includes, however, Mg^2+^-dependent RNA-cleaving 10–23 DNAzyme subunits^80^ that are pre-engineered to cleave the mRNA gene expressing the human early growth response-1 (EGR-1) protein. Moreover, the miR-155 triggered reconfiguration of CDN “N” to CDN “O” enriches the constituent GG_1_ thereby enhancing the cleavage rate of the EGR-1 mRNA. In addition, the miR-21-activated localized circuit leads to amplified release of the siRNA duplex s/as that is pre-engineered to cleave the hypoxia-inducible factor-1α (HIF-1α) mRNA. As the two proteins EGR-1 and HIF-1α are actively involved in tumor progression and metastasis,^81,82^ the cleavage of the EGR-1 mRNA and HIF-1α mRNA genes is anticipated to synergistically interfere with the gene expression, leading to bifunctional gene silencing and cell apoptosis. Accordingly, the operation of the localized circuit outlined in Figure 4A is anticipated to reveal important features for cancer-specific activation and synergistic therapeutic effect: (i) The miR-21-activated entropy-driven catalytic circuit provides an amplification route to use a low concentration of endogenous miRNA. (ii) The amplified release and assembly of the siRNA and DNAzyme products by the localized circuit leads to effective gene silencing. (iii) The miR-155 triggered reconfiguration of CDN “N” to CDN “O” leads to enhanced cleavage and silencing of the EGR-1 mRNA. The cooperative gene silencing of EGR-1 mRNA and HIF-1α mRNA leads to synergistic apoptosis of cells.

The performance of the *in vitro* model circuit shown in Figure 4A can be optically probed by FRET responses of the fluorophores Cy5/Cy3 as displayed in Figure 4B. The fluorescence spectra of the localized circuit before and after interaction with miR-21/miR-155 are displayed in panel I. In the absence of the miRs, an intense Cy3 fluorescence band and a low Cy5 fluorescence band are observed, indicating an inefficient FRET process between the fluorophores, curve a. In the presence of miR-21 or miR-155, the intensity of the Cy3 fluorescence band decreased, while the intensity of the fluorescence band of Cy5 reaches a medium level, curves b and c, indicating a medium level of the FRET process. In the presence of miR-21 and miR-155, an evident decrease in the Cy3 fluorescence band and concomitantly intensified Cy5 fluorescence band are observed, curve d, indicating an efficient FRET process. It should be noted that the depleted fluorescence intensity of Cy3 is higher than the evolved FRET intensity of Cy5. This phenomenon is well-established for the Cy3/Cy5 FRET pair and originates from two effects: (i) Cy3 exhibits a residual fluorescence intensity at λ = 666 nm, and thus, depletion of the fluorescence of Cy3 is accompanied by a lower overall fluorescence intensity of Cy5 at λ = 666 nm. (ii) The FRET efficiency is lower than 100% and, hence, the Cy5 evolved fluorescence intensity is lower. The medium FRET level of the circuit upon addition of miR-21 is attributed to the miR-21-triggered formation of CDN “N”, where the spatial proximity between Cy5 and Cy3 in constituent HH_1_ leads to the FRET signal. The intermediate level of the FRET signal upon subjecting the circuit to miR-155 is attributed to the cross-hybridization of single-strand domains present in H_1_ and H by complementary bases present in miR-155, leading to the spatial proximity between Cy5 and Cy3. The intense FRET process observed in the presence of miR-21 and miR-155 is attributed to the miR-155-triggered reconfiguration of CDN “N” to CDN “O” that stabilizes and upregulates constituent HH_1_. Figure 4B, panel II, presents the FRET results in the form of bar FRET intensities (*I*_666_/*I*_566_) as outputs, using miR-21 and miR-155 as inputs. Using the intermediate fluorescence intensity in the presence of miR-21 or miR-155 as threshold, the FRET output in the presence of miR-21 and miR-155 corresponds to an “AND” gate operation of the circuit (note that the threshold switching value of the AND gate could be enhanced by optimizing the concentrations of miRNA-21 and miRNA-155). The results demonstrated that the FRET fluorescence responses of the system could provide an optical sensing tool for analyzing the miRNAs.

In the next step, temporal FRET changes were used to probe the reaction kinetics and efficiency of the miRNA-responsive localized circuit. Particularly, the effects of spatial confinement of the core tetrahedron on the miRNA-stimulated circuit were addressed by following the temporal FRET process in the spatially localized circuit vs the temporal FRET process in a nonlocalized circuit consisting of diffusional, separated components without the tetrahedron component. Very low and slow FRET intensity changes are observed upon subjecting the nonlocalized circuit to miR-21 that yields diffusional CDN “N”, Figure 4B, panel III, curve b. In contrast, the temporal FRET changes upon subjecting the spatially localized circuit to miR-21 are depicted in Figure 4B, panel III, curve a. A rapid temporal FRET signal reaching a saturation value after ca. 20 min is observed, demonstrating the rapid entropy-driven formation of CDN “N”. A 70-fold enhanced kinetic rate and 13–14-fold enhanced hybridization efficiency are demonstrated. Similarly, subjecting the mixture of diffusional CDN “N” to miR-155 results in a substantially less efficient cross-hybridization process than in the presence of the spatially confined structure, Figure 4B, panel IV. The interhybridization rate is 10-fold enhanced, and the hybridization efficiency is 3.4-fold increased in the presence of the tetrahedra core unit, panel IV, curve a vs b (note that the initial rapid FRET signal in curve b, as compared to the slow evolution of the FRET signal by the dynamic reconfiguration of the circuit, panel III, is due to the availability of the intact constituent HH_1_ upon addition of miRNA-155). The results demonstrate that the dynamic processes in the spatially confined nanoenvironment reveal enhanced kinetics and efficiency, consistent with previous reports in related spatially confined systems.^15,83^ It should be noted that the primer-induced entropy-driven localized reconfiguration of the DNA-tetrahedral circuit T_2_ to CDN “N” reveals a low leakage phenomenon, often associated with strand displacement processes, resulting in a high signal-to-noise readout signal. This originates from optimized pre-engineering of the reaction circuits (for detailed experimental results and discussion, see Figure S11).

Taking advantage of the significantly improved hybridization efficiency and reaction kinetics by the spatial confinement effect, the spatially localized circuit is expected to enhance the sensing performance for detecting miR-21 and miR-155. As shown in Figure 4B, panel V, the fluorescence intensity ratios, *I*_666_/*I*_566_, are significantly intensified with the increase of the concentrations of miR-21. The logarithmic correlation between the fluorescence intensity ratio and miR-21 concentrations reveals a linear relationship within a 100 fM to 1 nM range with a detection limit of 30 fM (Figure 4B, panel VI). In contrast, a relatively low FRET intensity is observed for the nonlocalized circuit with a detection limit of 1 nM, which is 4 orders of magnitude higher than that of the localized circuit. These results indicate that spatial localization of the DNA circuit could effectively promote the sensing performance with exponential growth kinetics for ultrasensitive detection of miR-21, as compared to the nonlocalized DNA circuit. Figure 4B, panel VII, depicts the calibration curves corresponding to the fluorescence changes upon subjecting nonlocalized circuit, curve b, and the localized circuit, curve a, to variable concentrations of miR-155. The detection limit for analyzing miR-155 by the confined circuit corresponds to 30 pM, a 10-fold lower value as compared to that of nonlocalized circuit. Moreover, the selectivity of the confined circuit was also examined in the presence of other interfering miRs, Figure 4B, panel VIII. As expected, no distinct fluorescence responses were observed for foreign miRs. Clearly, the structurally confined circuit exhibited a significantly improved sensing performance for miR detection, compared to the nonlocalized circuit.

**Figure 5:**
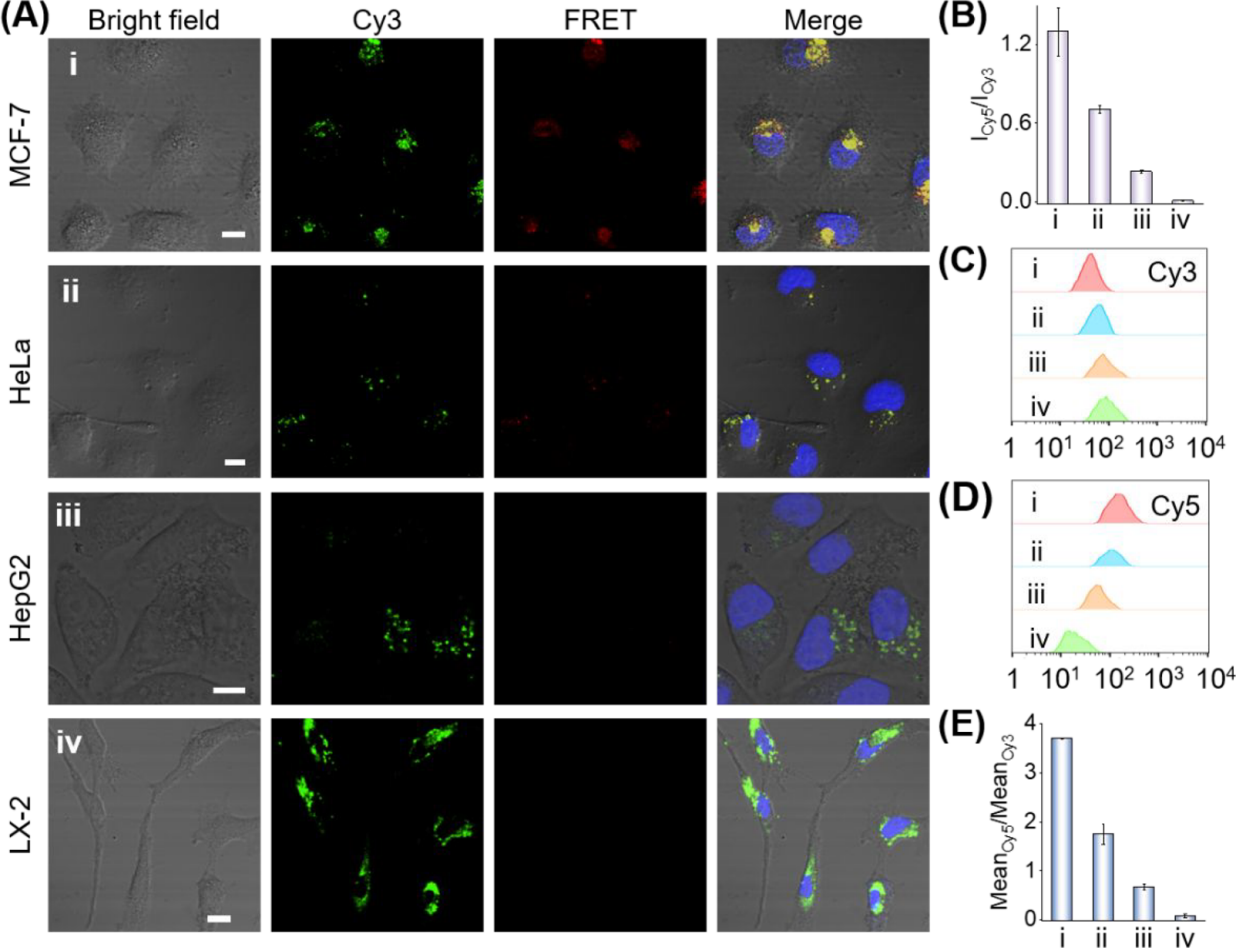
(A) Confocal fluorescence images of different cell lines subjected to the localized tetrahedron circuit T_2_, presented in Figure 4A: panel i, MCF-7 cells; panel ii, Hela cells, panel iii, HepG2 cells, and panel iv, LX-2 cells. Scale bar = 10 μm. (B) Integrated FRET intensities in the different cell lines expressed as *I*_666_(Cy5)/*I*_566_(Cy3). Data represent the analysis of 10 different frames of each cell line. Flow cytometry analysis of the different cell lines in panel A: (C) fluorescence of Cy3 in the different cell lines; (D) fluorescence of Cy5 in the different cell lines; (E) quantitative analysis of the flow cytometry fluorescent cell line samples represented as *I*_666_(Cy5)/*I*_566_(Cy3).

The advantages of the spatially localized circuit shown in Figure 4 for sensing the two miRNAs (miR-21 and miR-155) and the successful evolution and release of functional components for guided cleavage of two different mRNAs (expressing EGR-1 and HIF-1α) were then employed for theranostic applications in complex intracellular environments (leading to the synergistic apoptosis of cancer cells). The imaging capacity of the localized circuit was applied to discriminate four cell lines exhibiting different miR-21 and miR-155 expression profiles. The cell lines included MCF-7 breast cancer cells, exhibiting high expression of both miR-21 and miR-155, HeLa cervical cancer cells, exhibiting moderate expression of miR-21 and miR-155, HepG2 liver cancer cells overexpressing miR-21 only, and LX-2 normal liver cells with negligible expression levels of miR-21 or miR-155.^57,81^Figure 5A depicts the fluorescence confocal microscopy images of the cell lines treated with the localized circuit. The MCF-7 cells reveal intense red fluorescence corresponding to the Cy3/Cy5 FRET process, consistent with the high levels of miR-21/miR-155 in MCF-7, leading to the spatial proximity between Cy3/Cy5 generated by the constituent HH_1_. In contrast, the HeLa cells reveal moderate FRET intensities, consistent with the moderate expression of miR-21/miR-155. The HepG2 cells demonstrate a substantially lower FRET signal, consistent with the low expression of miR-155, and LX-2 cells do not show any FRET signal. Figure 5B depicts the integrated FRET signal intensities of the respective cell lines. The results follow the relative expressed contents of miR-21/miR-155 in the respective cells. The confocal fluorescence microscopy FRET results imaging the different cell lines are further supported by flow cytometry analysis of the cell lines treated with the localized circuit, Figure 5C–E. Figure 5C,D depict the flow cytometry analysis of Cy3 and Cy5 fluorescence, respectively, in the entire cell population. Figure 5E shows the mean ratio of the contents of the Cy5/Cy3 responsive cells that follow the FRET efficiencies in the respective cell lines. The flow cytometry results follow the confocal fluorescence microscopy results demonstrating different miR-21 and miR-155 expression profiles. In addition, the higher signal amplification efficiency and higher anti-interference performance of our localized circuit in living cells were also demonstrated by comparing with the nonlocalized circuit; see Figure S12 and accompanying discussion. The localized systems revealed a substantially higher fluorescence signal in MCF-7 cells, while the conventional nonlocalized system showed a relatively weaker fluorescence signal under the same conditions, demonstrating the high signal gain of the localized circuit for intracellular imaging.

**Figure 6:**
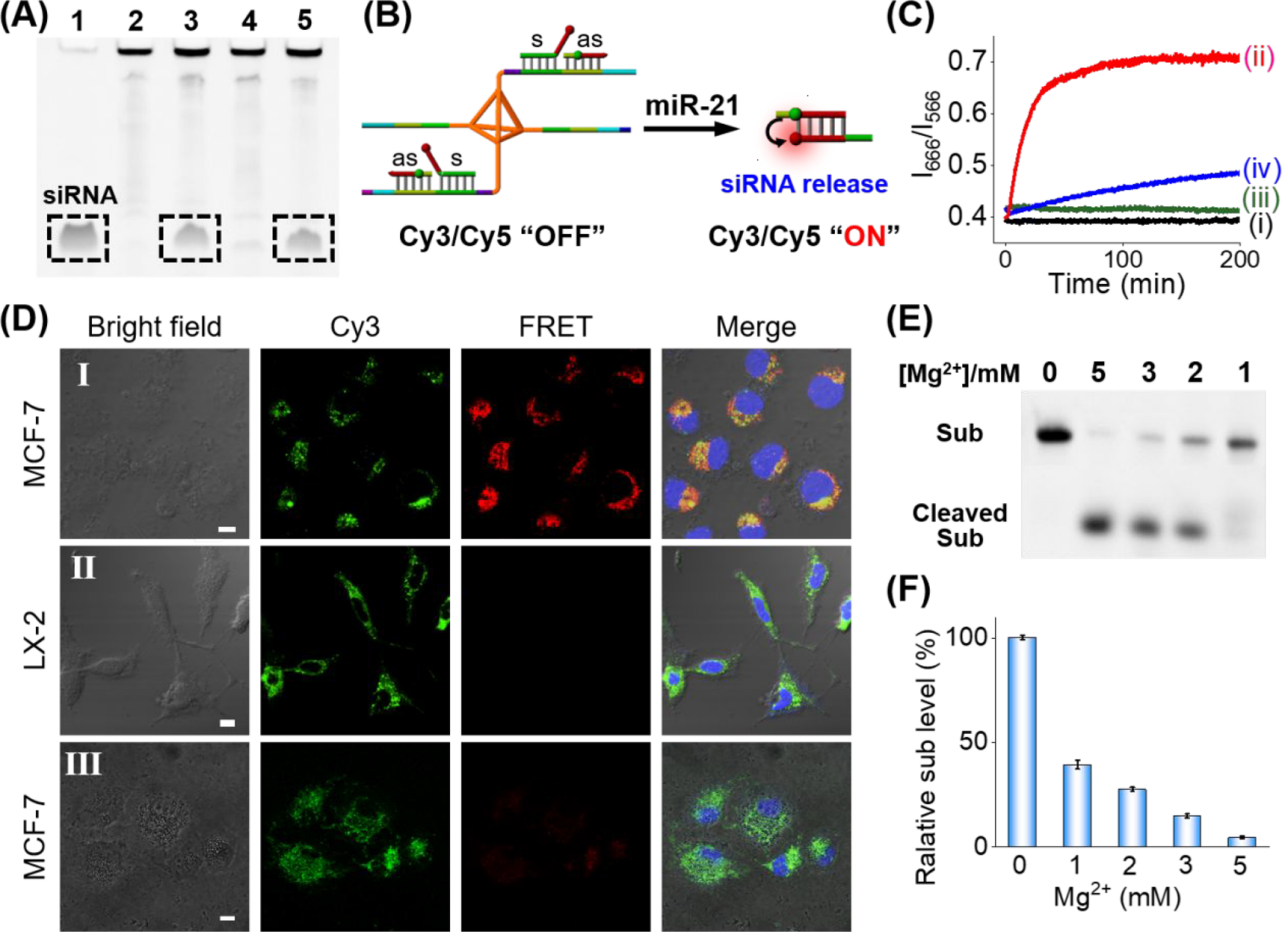
(A) Gel electrophoresis demonstrating the miR-21-guided release of the duplex siRNA, s/as, from localized DNA circuit: lane 1, reference siRNA s/as, 2 μM; lane 2, localized circuit; lane 3, localized circuit treated with miR-21; lane 4, localized circuit treated with miR-155; lane 5, localized circuit treated with miR-21 and miR-155. (B) Schematic probing of the miR-21-activated release of siRNA duplex s/as from the localized circuit. The s and as strands are labeled with Cy5 and Cy3, respectively. The resulting siRNA duplex leads to spatial proximity for enhanced FRET intensity. (C) Temporal FRET intensity changes expressed as *I*_666_/*I*_566_ corresponding to (i) the localized circuit in the absence of miR-21/miR-155; (ii) the localized circuit treated with miR-21; (iii) the localized circuit treated with miR-155; (iv) the control system consisting of diffusible components lacking the core tetrahedron, treated with miR-21. (D) Confocal fluorescence imaging corresponding to: panel I, MCF-7 cells treated with the localized circuit shown in panel B; panel II, LX-2 cells treated with the localized circuit shown in panel B; panel III, MCF-7 cells treated with the diffusible components without the core tetrahedron. (E) Gel electrophoresis image probing the efficiency of the cleavage of the EGR-1 mRNA by the Mg^2+^-ion dependent DNAzyme associated with constituent GG_1_ in the presence of variable concentrations of Mg^2+^ ion. (F) Quantitative evaluation of the yield of cleavage of the EGR-1 mRNA derived from panel E.

Next, the primary miR-21-triggered amplified release of the duplex siRNAs, s/as, from the localized circuit shown in Figure 4 was supported by gel electrophoresis and cell experiments. As depicted in Figure 6A, the band of siRNA duplex s/as is evident upon subjecting the localized circuit to miR-21, lane 3 vs lane 2, confirming that the miRNA-21 induced the release of the s/as duplex. The quantitative analysis of gel electrophoresis using ImageJ software indicates nearly complete release of siRNA (90%) from the miR-21-triggered localized circuit within 30 min, confirming the highly efficient release/assembly of siRNAs (Figure S13). Subjecting the localized circuit to miR-155 does not yield the s/as duplex, lane 4, yet treatment of the localized circuit with miR-21 and miR-155 shows the appearance of the duplex s/as. The miR-21 stimulated release of the duplex s/as was further supported by fluorescence measurement, Figure 6B,C. The s and as units were labeled with Cy5 and Cy3, respectively. While in the absence of miR-21 and miR-155, no FRET process between Cy5 and Cy3 is observed, curve i, a significantly intensified FRET signal is observed upon treatment of the localized circuit with miR-21, curve ii, indicating the separation of s and as components from the reaction module and assembly of a duplex s/as with spatial proximity between the FRET fluorescent probes. Control experiments show that no FRET signal is observed upon treatment of the localized circuit with miR-155, curve iii. Furthermore, subjecting the diffusible components without a tetrahedron core to the miR-21 leads to a very weak FRET signal, curve iv, confirming that the spatial confinement indeed leads to the quick and amplified release of the siRNA. Also, cell experiments utilizing the Cy5/Cy3 labeled localized circuit support the miR-21-stimulated release of the siRNA duplexes in the intracellular environment, Figure 6D and Figure S14. The MCF-7 cells with overexpressed miR-21 reveal significant FRET imaging signal after treatment with the localized circuit. In turn, the normal LX-2 cells treated with the localized circuit, do not show the FRET signals. The results demonstrate the sensitive and specific release capacity of the spatially localized circuit in response to intracellular miR-21. The control experiment, employing the nonlocalized system, reveals a substantially lower fluorescence signal, Figure 6D, panel III. The results demonstrate that the spatially localized circuit indeed induces efficient release of the target siRNA drugs in the cytoplasm, which is essential for effective gene silencing.^84^ In addition, the catalytic activities of the DNAzyme-functionalized constituent GG_1_ (cf. Figure 4) toward the cleavage of EGR-1 mRNA were probed by gel electrophoresis. Figure 6E shows the effects of Mg^2+^ ion concentrations on the cleavage efficiency of EGR-1 mRNA, where Figure 6F shows the quantitative cleavage efficiency of the mRNA in the presence of variable concentrations of Mg^2+^ ion. As the concentration of the Mg^2+^-ion is lower, the cleavage efficiency is lower. The results show, however, that in the concentration range 1–3 mM of Mg^2+^ ion, that is present in cells,^85^ the cleavage efficiency of the mRNA is visible, indicating the possible application of the DNAzyme as an EGR-1 gene silencing catalyst. The longer incubation time led to a higher cleavage efficiency (Figure S15). In addition, we used gel electrophoresis to compare the stability of the siRNA-functionalized tetrahedra and naked siRNA in culture medium containing 10% fetal bovine serum at 37 °C (Figure S16). Naked siRNAs exhibited evident degradation by ca. 70% after 4 h, while siRNA-functionalized tetrahedra show degradation of only 17% after 12 h and 33% after 24 h, indicating a higher stability of the siRNA-functionalized tetrahedra against nuclease degradation.

**Figure 7:**
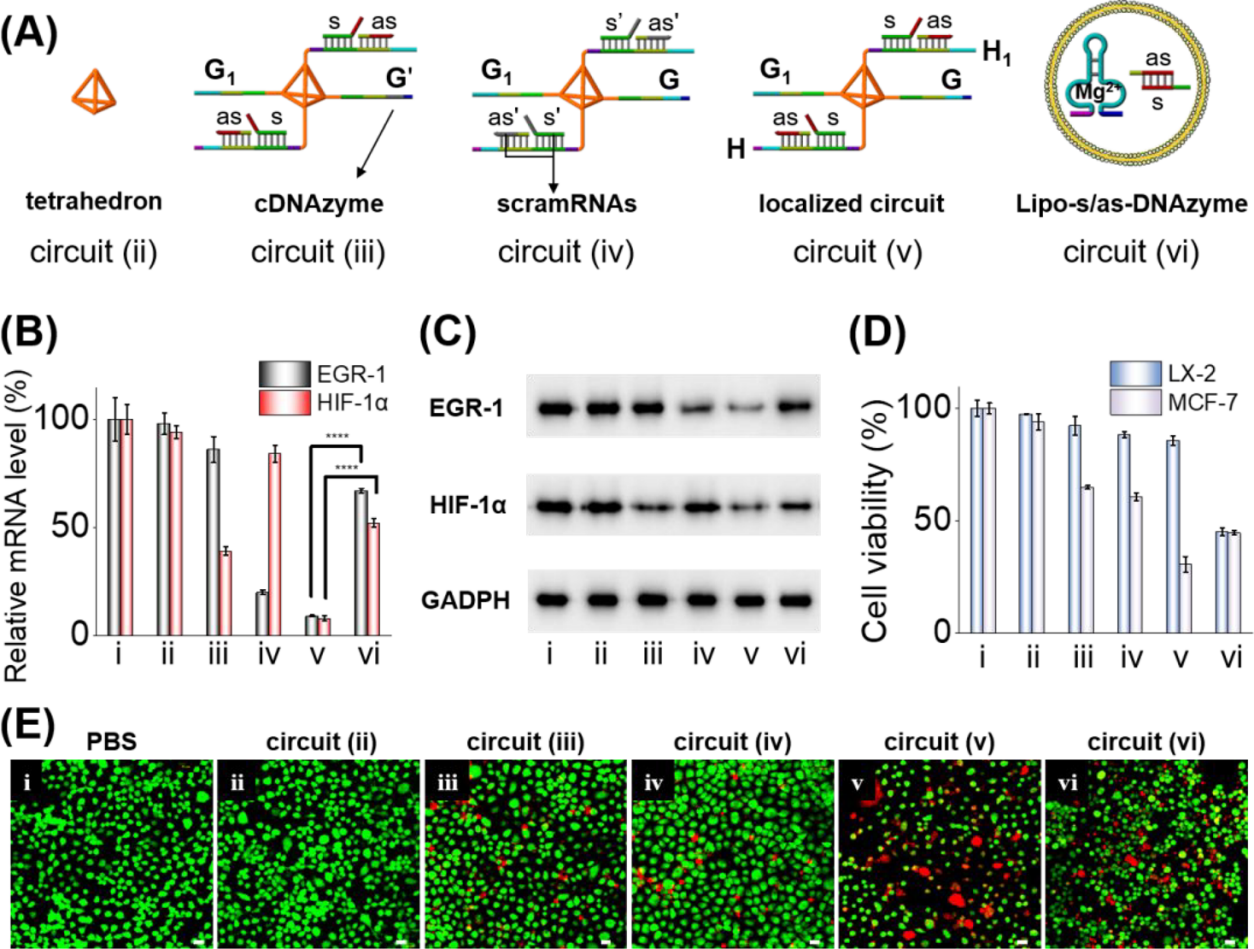
(A) The intact localized circuit and several control systems were employed to probe the expression levels of the mRNAs and proteins of EGR-1/HIF-1α, and the cell viabilities of LX-2/MCF-7 cells. (B) Relative expression yields of EGR-1/HIF-1α mRNAs monitored by RT-qPCR in MCF-7 cells treated with the different circuits: (i) PBS; (ii–vi) detailed in panel A. (C) Western blot images corresponding to the expression levels of proteins EGR-1/HIF-1α and control GADPH in MCF-7 cells subjected to different circuits: (i) PBS; (ii–vi) detailed in panel A. (D) Relative cell viability of LX-2 and MCF-7 cells subjected to the different circuits: (i) PBS; (ii–vi) detailed in panel A. (E) Confocal fluorescence microscopy images corresponding to MCF-7 cells treated with (i) PBS and (ii–vi) detailed in panel A. Green stained cells correspond to living cells; red stained cells correspond to dead cells.

Following the successful demonstration of the efficient *in vitro* release/assembly of siRNA and DNAzyme by the miR-21-activated localized circuit, we evaluated the *in vitro* gene silencing capacities of the localized circuit for antitumor therapy. To allow a comparison of the gene silencing efficacies of the localized circuit, we designed several control systems to be adapted in the cell experiments, and these are summarized in Figure 7A. The control systems include pure buffer (i), the bare tetrahedron unit (ii), the s/as-functionalized localized circuit modified with control DNAzyme (cDNAzyme) in a new tether G′ by substituting one nucleotide of the DNAzyme catalytic core (iii), the scrambled siRNA sequences, s′/as′-functionalized localized circuit modified with GG_1_ tethers generating the active DNAzyme (iv), the integrated localized circuit that includes the s/as components and the DNAzyme (v), and a commercial transfection system (liposome + DNAzymes + siRNAs) (vi). The localized circuit and the control circuits were subjected to MCF-7 cancer cells to elucidate the functions of the constituents on gene silencing efficacies. The relative mRNA expression levels of HIF-1α and EGR-1 in MCF-7 cells were investigated by using real-time quantitative polymerase chain reaction (RT-qPCR) assay (Figure 7B). The expression of HIF-1α is inhibited by systems iii and v (61% and 92%, respectively), indicating that the siRNAs are active in MCF-7 cancer cells to efficiently cleave the target mRNAs for gene silencing. Also, the expression of EGR-1 mRNA is inhibited by the systems iv and v, confirming the existence of the active DNAzyme GG_1_ in MCF-7 cells (inhibition by 80% and 91%). In turn, a commercial transfection system (liposome + DNAzyme + siRNAs) shows moderate inhibition in the expression of the mRNAs of HIF-1α and EGR-1 (48% and 33%), demonstrating the advantages of the localized circuits for gene silencing. Compared with the single-gene therapy, the combined bis-gene therapy reveals significantly enhanced downregulation of the EGR-1/HIF-1α tumor gene and protein expression, demonstrating the cooperatively enhanced gene-silencing abilities of HIF-1α and EGR-1. Moreover, the protein expression levels in the MCF-7 cells subjected to the localized circuit and the other control circuits were evaluated, Figure 7C. Evidently, the expression of the EGR-1 is inhibited in the cells treated with iv and v, consistent with the generation of the DNAzyme by these circuits that cleaved the EGR-1 mRNA. Also, the expression of HIF-1α is inhibited in the cells treated with iii and v, consistent with the release of the siRNA s/as duplex in these circuits. Furthermore, the reference expression of GADPH in the cell is not affected by any of the circuits, as demonstrated by the Western blot images. After the successful control of the mRNA and protein expression, the cytotoxicity of the circuits toward the MCF-7 cancer cells and LX-2 normal cells was assessed with the different circuits i–vi, presented in Figure 7D. Evidently, the pure buffer (i) and bare tetrahedron (ii) did not have any cytotoxic effect on the cells. The structures iii and iv had only moderate cytotoxic effect on the MCF-7 cells and no effect on the LX-2 cells. The structure v demonstrating dual gene silencing effects revealed ca. 70% cell death of MCF-7 and almost no cytotoxic effect on the LX-2 cells, consistent with the cooperative gene silencing functions. The liposome systems loaded with the DNAzyme and siRNAs reveal about 50% cell death toward both the MCF-7 and LX-2 cells. Thus, substantially enhanced and selective therapeutic efficacy of the localized circuit as compared to liposome systems is observed. Moreover, the live/dead cell analysis in MCF-7 cells, Figure 7E, also demonstrates that the cell apoptosis by the dual synergistic gene silencing pathways involving the HIF-1α mRNA and EGR-1 mRNA cleavage routes provided by the localized circuit is substantially enhanced as compared to the apoptosis induced by the individual HIF-1α or EGR gene silencing units.

**Figure 8:**
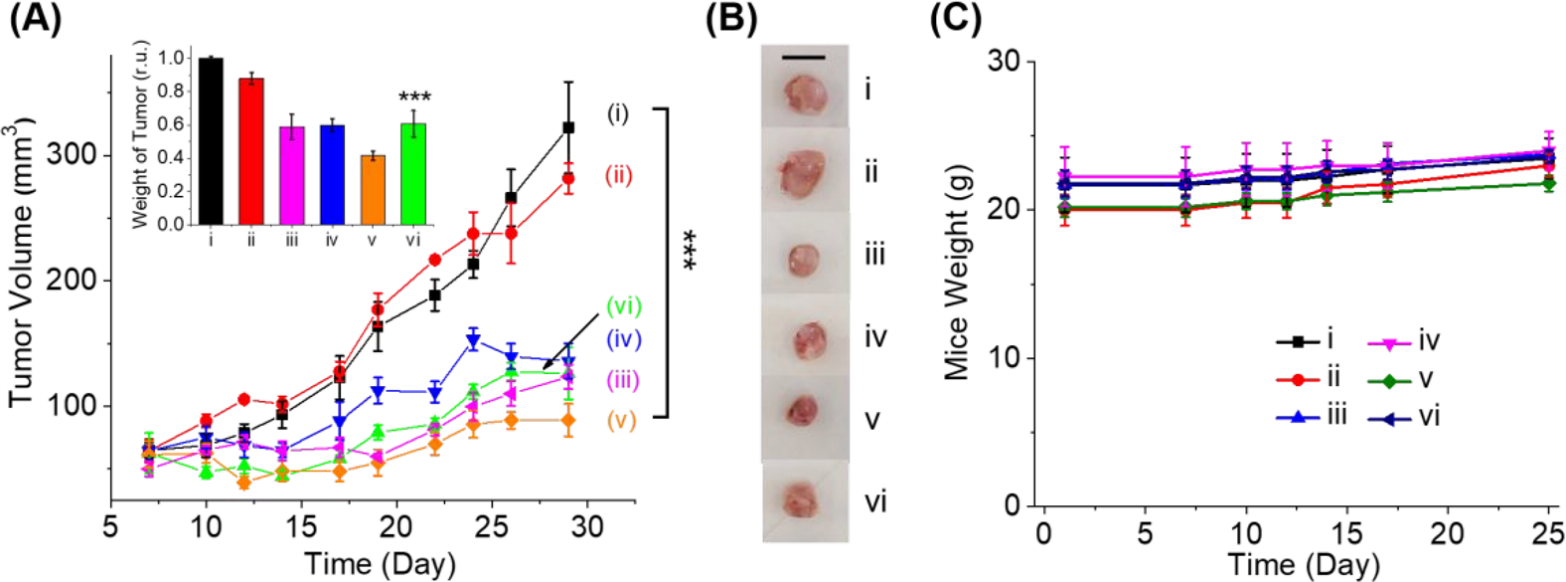
(A) Time-dependent tumor volume growth profiles in the presence of (i) pure buffer and (ii–vi) circuits detailed in Figure 7A. Inset: tumor volume changes, using different circuits in the form of a bar presentation. (B) Tumor images after treatment with the respective circuits; scale bar = 1 cm. (C) Body weight of mice treated with different circuits. All results are presented as mean ± SEM. Significant results were evaluated using *t* test; ***P < 0.001.

In the next step, the preliminary *in vivo* effect of the localized DNA circuit on the apoptosis of cancer cells and inhibition of tumor growth was evaluated. While facing difficulties in eliciting MCF-7 tumors in mice, we successfully developed MDA-MB-231 breast cancer tumors in xenograft mice. Since the MDA-MB-231 cells include overexpressed miR-21 and miR-155, the effect of intratumor (IT) injection of the intact localized circuit v revealing the synergistic cooperative silencing mRNA effect on cell apoptosis and of circuits iii and iv revealing individual HIF-1α mRNA or EGR-1 mRNA silencing effects on the growth of the tumors were examined. A further control system included the evaluation of the effects of IT treatment of the tumors with bare tetrahedra (circuit ii) or with transfected carriers loaded with the siRNA agents and the DNAzyme constituents (circuit vi), Figure 8A. Figure 8A depicts the temporal tumor volume growth profiles upon IT treatment with the different circuits and the control systems (For details on the experiments, see page S8, Supporting Information). The tumor bearing mice treated with the intact localized tetrahedra circuit v revealing cooperative HIF-1α/EGR-1 mRNA silencing effect did not show any temporal volume changes over 4 weeks, indicating that the growth of the tumors was fully inhibited, curve v. In turn, the tumors treated with circuits iii or iv, exhibiting a single pathway to silence the HIF-1α mRNA or EGR-1 mRNA, revealed a 65–70% inhibition of growth of the tumors, curves iii or iv, as compared to the volume growth by the reference buffer and bare tetrahedra systems, curves i and ii. For comparison, the effect of IT tumor treatment with liposome-transfected siRNA/DNAzyme (circuit vi) on tumor growth profile is depicted in curve vi. The inhibition of the growth of the tumors by the different tetrahedral agents followed the effect of the agents on the viability of the breast cancer cells by the agents (Figure 7). In addition, histopathological evaluation of the tumor tissues treated with the different therapeutic circuits (iii–vi) and control modules consisting of the inert tetrahedra (ii) and pure buffer (i), shown in Figure 7A were performed. The results are presented in Figure S17A and accompanying discussion in Supporting Information. The histopathological experiments following the degree of apoptotic (dead) cells further confirm the cell experiments and *in vivo* IT-treated tumor growth inhibition studies by different circuits. While the treatment of the tumors with circuits iii or iv, inducing the silencing of HIF-1α mRNA or EGR-1 mRNA only, led to a moderate degree of apoptotic cells, subjecting the tumors to the cooperatively operating circuit (v), led to a 2-fold enhanced degree of apoptotic cells, Figure S17B. Also, no apoptotic effect was observed on the tumors treated with the control systems (i) pure buffer and (ii) inert DNA tetrahedra, suggesting that the apoptotic effects, indeed, originate from the guided silencing of the mRNAs, by the respective circuits. Moreover, it should be noted that no weight losses of the average weights of the mice treated with the different circuits, along the experiments, were observed, Figure 8C, implying that the different circuits are nontoxic.

## Conclusion

This study introduced the fundamental concept of primer-guided, high-throughput, entropy-driven evolution of DNA-based CDNs. The entropy gain associated with the circuit provides a new catalytic principle for driving the emergence of the CDNs. The amplified, high-throughput, and cascaded capacities of the entropy-driven circuits for the emergence of CDNs were demonstrated. The concept was then applied to develop a programmable DNA circuit for the effective *in vitro* and *in vivo* spatially localized theranostic, gene-regulated treatment of cancer cells. A DNA tetrahedron core unit was functionalized at its corners with four engineered tethers, where two of the tethers were modified with two caging siRNA subunits, and the tethers were encoded with engineered sequences providing the capacity to emerge into a functional [2 × 2] CDN, modified with DNAzyme units. While the core tetrahedron unit provides an effective vehicle for cell permeation of the reaction module, the structural confinement of the functional tethers associated with the tetrahedron core enables the fast miRNA-21-stimulated release of the siRNA and the assembly of the mRNA-cleaving DNAzyme. The cooperative mRNA silencing pathways lead to the selective and effective apoptosis of MCF-7 cancer cells. Moreover, the study demonstrated significant concepts for effective gene therapy. The tetrahedral core module to assemble the gene therapeutic circuit revealed several advances, including enhanced and effective cell permeation, efficient spatially localized reconfiguration of the constitutional dynamic circuit, leading to the cooperative gene silencing mechanism, effective selective apoptosis of cancer cells, and the translation of an *in vitro* medical treatment concept into an *in vivo* practice. Particularly, the biocompatibility of the DNA tetrahedral circuit and its versatile applicability are noteworthy. Beyond demonstrating the unprecedented use of programmed entropy-driven circuits stimulating biological responses in cells and contribution of the principles to the development of biosensors, biomedicine, and Systems Chemistry, broader impacts of the concepts may be envisaged: (i) The triggered release of other functional nucleic acids by the entropy-driven circuit, such as ribozymes, antisense oligonucleotides, or splicing DNAzymes as control units of gene/protein expression pathways, may be designed.^78,86^ Also, aptamers could be linked to the reaction module and activate the circuit by aptamer/ligand complexes.^17^ (ii) The study demonstrated the primer-induced entropy-driven cascaded emergence of two CDNs. Accordingly, by applying seesaw gates,^12,36^ the primer-induced cascaded evolution of intercommunicating scalable, higher-order CDNs of enhanced complexity and programmable hierarchical functionalities may be envisaged. Particularly, photoresponsive caged hairpin structures have been recently applied for the spatiotemporal programmed synthesis of DNA nanostructures and machines.^87,88^ By tethering such photoresponsive hairpins to core DNA tetrahedral structures, functional reaction module revealing light-triggered spatiotemporal theranostic applications may be realized. (iii) The integration of dynamic networks and circuits into cell-like containments (protocells) attracts substantial recent research efforts.^89,90^ The assembly of dose-controlled multiconstituent systems into such carriers is, however, a challenge. The integration of intact supramolecular multiconstituent tetrahedron-based frameworks in cell-like containments^91,92^ and their activation by auxiliary triggers, e.g., primer-induced entropy-driven pathways, could provide means to stimulate diverse chemical transformation for different applications. (iv) Moreover, the concept of entropy-driven mechanism introduced in our study presents a route to evolve network of enhanced complexities and functionalities from simple nucleic acid building blocks and thus provides insight for the evolution of biological networks under prebiotic conditions.

## Supporting Information Available

Additional experimental details, materials, DNA sequence, methods, characterizations, calibration curves, gel electrophoretic images, optimization of the reaction module, histopathological evaluation, and tables including the concentrations of the constituents associated with the respective CDNs (PDF)
