# Supplementary material for: Spatially Localized Entropy-Driven Evolution of Nucleic Acid-Based Constitutional Dynamic Networks for Intracellular Imaging and Spatiotemporal Programmable Gene Therapy
Source: J Am Chem Soc. 2024 Jul 16;146(30):20685–99. doi: 10.1021/jacs.4c03651 (PMC11295181; doi:10.1021/jacs.4c03651)
Supplement: Supplementary file 1 — ja4c03651_si_001.pdf [file ja4c03651_si_001.pdf]

Supporting Information

Spatially Localized Entropy-Driven Evolution of Nucleic Acid-Based Constitutional Dynamic Networks for Intracellular Imaging and Spatiotemporal Programmable Gene Therapy

Nina Lin,[†] Yu Ouyang,[‡] Yunlong Qin,[‡] Ola Karmi,[§] Yang Sung Sohn,[§] Songqin Liu,[†] Rachel Nechushtai,[§] Yuanjian Zhang,[†,*] Itamar Willner[‡,*] and Zhixin Zhou[†,*]

[†]School of Chemistry and Chemical Engineering, Southeast University, Nanjing 211189, China

[‡]Institute of Chemistry, The Hebrew University of Jerusalem, Jerusalem 91904, Israel

[§]Institute of Life Science, The Hebrew University of Jerusalem, Jerusalem 91904, Israel

*Email: Yuanjian.zhang@seu.edu.cn

*Email: willnea@vms.huji.ac.il

*Email: zhixin.zhou@seu.edu.cn

Experiment Section

Materials. Tris-Acetate-EDTA (TAE) buffer solution, DNA Marker A (25-500 bp), and "GelRed nucleic acid gel stain" were purchased from Sangon Biotech Co., Ltd. (Shanghai, China). Agarose was purchased from Aladdin Reagent Co., Ltd. (China). Polyacrylamide gel electrophoresis (PAGE) Gel Fast Preparation Kits (15%) were purchased from Shanghai Epizyme Biomedical Technology Co., Ltd (China). Dulbecco's modified Eagle's medium (DMEM), foetal bovine serum (FBS), trypsin, phosphate buffered saline (PBS), fluorescein diacetate (FDA), propidium iodide (PI), and Hoechst 33342 were all obtained from KeyGen Biotech. Co. Ltd. (Nanjing, China). Ultrapure water (resistance > 18 MΩ·cm) was used in all experiments. All DNA and RNA oligonucleotides were synthesized by Sangon Biotech Co., Ltd.

The oligonucleic acid sequences used in the study include (from 5' end to 3' end):

A: *TGGAGACGTAGGGTATTGAATG*AGGGCCGTAAGTTAGTTGGAGACGTAGGCACCCATGTTACTCT

A_1 (fuel): GATATCAGCGATCCTACGTCTCCAACTAACTTACGGCCCTCATTCAATACCCTACG

B: *TGGAGACGTAGGGTATTGAATG*AGGGCCGTAAGTTAGTTGGAGACGTAGGCACCCATGTTCGTCA

B_1 (fuel): CTGCTCAGCGATCCTACGTCTCCAACTAACTTACGGCCCTCATTCAATACCCTACG

P_1/P_4: *CATTCAATACCCTACGTCTCCA*

M_1/M_4: CCCTCATTCAATACCCTACG

M_2: CCTACGTCTCCAACTAACTTACGG

C: *TGGAGACGTAGGGTGAAAGTAG*TGTC*GGCTAATAAGTATGGAGACGTAGG*CACCCATGTTTCAGT

C_1 (fuel): CTGTTCAGCGATCCTACGTCTCCATACTTATTAGCCGACACTACTTTCACCCTACG

D: *TGGAGACGTAGGGTGAAAGTAG*TGTC*GGCTAATAAGTATGGAGACGTAGG*CACCCATGTTCCTGA

D_1 (fuel): GTCCTCAGCGATCCTACGTCTCCATACTTATTAGCCGACACTACTTTCACCCTACG

P_2: *CTACTTTCACCCTACGTCTCCA*

P_3: *CATTCAATACCCTACGTCTCCA*TACTTATTAGCC

M_3: GACACTACTTTCACCCTACGTC

Threshold: AAGTATGGAGACGTAGGGTATTGAATGAG

E: AGAATCTGATAGAGGTAGAGTGAT*TGGAGACGTAGGGTATTGAATG*AGGGCCGTAAGTTAGTTGGAGACGTAGGCACCCATGTTACTCT

E_1 (fuel): CTGCTCAGCGATCCTACGTCTCCAACTAACTTACGGCCCTCATTCA

ATACCCTACG GGCAGACCAGAGTCTTGTACCAGT
F: CTTACGTAGTTAGTATGCTTGCTGT*TGGAGACGTAGGGTATTGAATG*AGGG CCGTAAGTTAGTTGGAGACGTAGG<u>CACCCAT*GT*TCGTCA</u>
F_1 (fuel): <u>GATATCAGCGAT</u>CCTACGTCTCCAACTAACTTACGGCCCTCATTC AATACCCTACG AAATGTAGTGCTTACGTCAGGCAG
M_5: TGCCTACGTCTCCAACTAACTTACGG
G_1: AGAATCTGATAGAGGTAGAGTGAT*TCAACATCAGTCTGATAAGCTA*AGGG CCGTAAGTTAGTTGGAACATGAGGAAATGAGAGCCTCTGACGAGCA
G (fuel): CCGCGGCCAGGCTAGCTACAACGACCTGAGAGGCCCTCATGTTCC AACTAACTTACGGCCCTTAGCTTATCAGACTGAATTTTGCTTACGTCAGGCA G
G-C (fuel): CCGCGGCCAGGCTACCTACAACGACCTGAGAGGCCCTCATGTT CCAACTAACTTACGGCCCTTAGCTTATCAGACTGAATTTTGCTTACGTCAGG CAG
H_1: CTTACGTAGTTAGTATGCTTGCTTT*TCAACATCAGTCTGATAAGCTA*AGGG CCGTAAGTTAGTTGGAACATGA-Cy3-GGAAATGAGAGCCTCTGATTAGC ATTAA
H_1-C: CTTACGTAGTTAGTATGCTTGCTTT*TCAACATCAGTCTGATAAGCTA*A GGGCCGTAAGTTAGTTGGAACATGAGGAAATGAGAGCCTCTGATTAGCATT AA
H (fuel): ACCCCTATCACTTAGAGGCCCT-Cy5-CATGTTCCAACTAACTTACG GCCCTTAGCTTATCAGACTGAATTTTAGAGTCTTGTACCAGT
H-C (fuel): ACCCCTATCACTTAGAGGCCCTCATGTTCCAACTAACTTACG GCCCTTAGCTTATCAGACTGAATTTTAGAGTCTTGTACCAGT
s: CATGAGGAAATGAGAGAAATTCCCTTAGCTTATCAGACTGAT
as: TTTCTCTCATTTCCTCATGTTCCAACTAACTTACGG
s-Cy5: Cy5-CATGAGGAAATGAGAGAAATTCCCTTAGCTTATCAGACTGAT
as-Cy3: TTTCTCTCATTTCCTCATG-Cy3-TTCCAACTAACTTACGG
Helper: TTTCTCTCATTTC
EGR-1 substrate: GCCTGCTCGTCCAGGrArUGGCCGCGGCCAAGG
S_{G1}: CACTCTACCTCTATCAGATTCTTTTACATTCCTAAGTCTGAAACATTAC AGCTTGCTACACGAGAAGAGCCGCCATAGTA
S_{H1}: GCAAGCATACTAACTACGTAAGTATTATCACCAGGCAGTTGACAGTG TAGCAAGCTGTAATAGATGCGAGGGTCCAATAC
S_G (Fuel): TCAACTGCCTGGTGATAAAACGACACTACGTGGGAATCTACTATG GCGGCTCTTCTTTCTGCCTGACGTAAGC
S_H (Fuel): TTCAGACTTAGGAATGTGCTTCCCACGTAGTGTCGTTTGTATTGG ACCCTCGCATTTTACTGGTACAAGACTC
miRNA-21: UrArGrCUUrAUrCrArGrArCUrGrAUrGUUrGrA
miRNA-155: UUrArAUrGrCUrArAUrCrGUrGrAUrArGrGrGrGU
miRNA-221: rArGrCUrArCrAUUrGUrCUrGrCUrGrGrGUUUrC
miRNA-16: UrArGrCrArGrCrArCrGUrArArAUrAUUrGrGrCrG
miRNA let-7a: UrGrArGrGUrArGUrArGrGUUrGUrAUrArGUU
miRNA let-7b: UrGrArGrGUrArGUrArGrGUUrGUrGUrGrGUU

siRNA(s): rCrAUrGrArGrGrArArAUrGrArGrArGrArArAdTdTrCrCrCUUrArGrCU UrAUrCrArGrArCUrGrAU

siRNA(as): UUUrCUrCUrCUrCrAUUUrCrCUrCrAUrGdTdTrCrCrArArCUrArArCUUr ArCrGrG

For gel:

[2×2]CDN

A-gel: TGGAGACGTAGGGTATTGAATGAGGGCCGTAAGTTAGTTGGAG ACGTAGGCACCCATGT

A_1 (fuel)-gel: CCTACGTCTCCAACTAACTTACGGCCCTCATTCAATACCCTA CG

M_1-gel: CCCTCATTCAATACCCTACGCCACATACATCATATTTTTTTTTTTT TTTTTTTTTT

Cascade-CDN

A-gel-1: TGGAGACGTAGGGTATTGAATGAGGGCCGTAAGTTAGTTGGAGA CGTAGG

A_1 (fuel)-gel: CCTACGTCTCCAACTAACTTACGGCCCTCATTCAATACCCTA CG

B-gel: TGGAGACGTAGGGTATTGAATGAGGGCCGTAAGTTAGTTGGAGAC GTAGGCACCCA

M_1-gel-1: CCCTCATTCAATACCCTACGCCACATACATCATATTTTTTTTTT

C-gel: TGGAGACGTAGGGTGAAAGTAGTGTCGGCTAATAAGTATGGAG ACGTAGGCACCCATGTTTCAGTTTTTTTTTTTTTTTTTTTT

C_1 (fuel)-gel: TTTTTTTTTTTTTTTTTTTTTTTTTTCTGTTCAGCGATCCTACG TCTCCATACTTATTAGCCGACACTACTTTCACCCTACG

D_1 (fuel)-gel: TTTTTTTTTTTTTTTTGTCCTCAGCGATCCTACGTCTCCATACT TATTAGCCGACACTACTTTCACCCTACG

M_3-gel: GACACTACTTTCACCCTACGTCATTCTTCTCTCTCTCTCTCTCTCT CTCTCTCTCTCTCTCTCTCTCTCTCT

Tetrahedra-CDN

E-gel: AGAATCTGATAGAGGTAGAGTGATTGGAGACGTAGGGTATTGAAT GAGGGCCGTAAGTTAGTTGGAGACGTAGGCACCCATG

F_1-gel: CCTACGTCTCCAACTAACTTACGGCCCTCATTCAATACCCTACGA AATGTAGTGCTTACGTCAGGCAG

Substrate sequences for DNAzyme subunits associated with consitituents:

Sub 1 (AA_1/EF_1): ROX-AGAGTATrAGGATATC-BHQ2

Sub 2 (BB_1/FE_1): FAM-TGACGATrAGGAGCAG-BHQ1

Sub 3 (AB_1/EE_1): Cy5-AGAGTATrAGGAGCAG-BHQ2

Sub 4 (BA_1/FF_1): FAM-TGACGATrAGGATATC- BHQ1

Sub 5 (CC_1): FAM-ACTGAATrAGGAACAG-BHQ1

Sub 6 (DD_1): ROX-TCAGGATrAGGAGGAC-BHQ2

Sub 7 (CD_1): Cy5-ACTGAATrAGGAGGAC-BHQ2

Sub 8 (DC_1): FAM-TCAGGATrAGGAACAG-BHQ1

The respective DNAzyme subunit sequences in each of oligonucleic acid sequence.

The sequences for recognition of primer (P_1, P_2, P_3 and P_4) are italic.

Measurements. Fluorescence spectra were recorded with a Cary Eclipse Fluorometer (Agilent Technologies). The excitation of FAM, ROX, and Cy5 was performed at 496 nm, 588 nm, and 648 nm, respectively, while the emission of FAM, ROX, and Cy5 was recorded at 516 nm, 608 nm, and 668 nm, respectively. The FRET fluorescence spectra between Cy3 and Cy5 were collected from 540 to 800 nm with excitation wavelength at 520 nm. UV/vis spectra were performed on a Cary 60 UV/Vis spectrometer (Agilent Technologies). The PAGE and agarose gels were run on VE-180 and HE-120 electrophoresis units, respectively (Tanon, China). The fluorescence of tetrahedra in cells was monitored with the Olympus FluoViewTM FV3000 confocal laser-scanning microscope, and all images were analyzed with image J.

Emergence of various DNA-based constitutional dynamic networks (CDNs) by entropy-driven catalytic DNA circuits. The entropy-driven DNA circuits were operated in 1 × PB buffer containing 10 mM NaH_2PO_4/Na_2HPO_4 (pH 7.0) and 10 mM $MgCl_2$, unless otherwise specificized.

For emergence of a [2×2] CDN "K" composed of AA_1, AB_1, BA_1 and BB_1, substrate and fuel strands, S_1, S_2, A_1, and B_1, 1 μM each, were mixed, and then different concentrations of the primer P_1 were subjected to the mixture, followed by incubating at 25 °C for 1 hour. The control experiment was performed under the same condition yet in the absence of P_1.

For cascaded emergence of CDN "L" and CDN "K", substrate and fuel strands, S_1, S_2, S_3, S_4, A_1, B_1, C_1, and D_1, 1 μM each, were mixed, and then the primer P_2, 1 μM, was subjected to the mixture followed by incubating at 25 °C for 3 hours. The control experiment was performed under the same condition yet in the absence of P_2.

For emergence of CDN "M" attached on tetrahedra, the mixtures of S_E, S_{E1}, S_F, S_{F1}, E_1, and F_1, 1 μM each, were heated at 95 °C for 5 min and cooled to 4 °C within 1 min. Subsequently, 1.5 μM S_5, and S_6, were incubated with the as-prepared E_1-/F_1-functionalized tetrahedron at 30 °C for 1 hour followed by purifying by ultrafiltration (100kDa molecular weight cutoff) to remove the excessive S_5, and S_6 remaining in

solution. The primer P_4, 1 μM, was subjected to the mixture followed by incubating at 25 °C for 3 hours. The control experiment was performed under the same condition yet in the absence of P_4.

Fluorescence Assay. For detection of miRNAs using localized circuit, different concentrations of miRNA-21 and miRNA-155 were added to 100 μL of 1 × PB buffer solution containing 100 nM tetrahedron sensing module, followed by incubation for 5 hours at room temperature. The FRET fluorescence spectra between Cy3 and Cy5 were collected from 540 to 800 nm with excitation wavelength at 520 nm.

Cell culture. Human breast cancer cells (MCF-7) were grown in 5% CO_2 DMEM medium supplemented with 10% FBS and 1% antibiotics (KeyGEN BioTECH, China). Human liver cancer cells (HepG2) were grown in 5% CO_2 DMEM medium supplemented with 10% FBS and antibiotics (KeyGEN BioTECH, China). Cells were planted one day prior to the experiment on μ-slide 4 well glass bottom for confocal microscopy.

Confocal microscopy measurements. For cell imaging experiments, one day prior to the experiment, cells were planted in μ-slide 4 well glass bottom. Cells were incubated with the different systems after washing with PBS. The different systems (100 nM) were incubated with cells for 10 hours and then washed with DMEM twice and replenished with the fresh medium for the measurement. An external 561 nm excitation with an accompanying emission ranging from 570 to 620 nm was selected for the green channel of fluorophore (Cy3) donor. The external 561 nm FRET stimulation with an accompanying emission signal collection ranging from 650 to 700 nm was selected for the yellow channel of fluorophore (Cy5) acceptor. To achieve a reliable FRET readout, the background FRET signal, originating from solely Cy3/Cy5 fluorophore, was subtracted from each of the samples.

In vitro cytotoxicity and therapeutic study. MCF-7 cells were seeded into 96-well plate at the density of ca. 1×10^5 cells per well and incubated overnight under hypoxia

environment. Then, the different formats of the circuits were incubated with MCF-7 cells for 12 h. The cells were washed three times with PBS to remove the excessive circuits, and 10 μL of CCK-8 solution and 100uL of DEME was added into each well following by a further 2 h incubation. Finally, the absorbance at 450 nm (OD 450) of the wells was measured with a microplate reader (Thermo Scientific).

MDA-MB-231 Cells were seeded in a 96-well plate at a density of 1.5 × 10^5 and allowed to culture for overnight. The different circuits were incubated with cells for 12 hours. Cells were washed two times with DMEM-HEPES to remove the excessive circuits and Cell viability was determined using the fluorescent redox probe, Presto-blue™ (Invitrogen™, A13261). Cells are incubated with Presto-blue for 1 hour and Presto-blue fluorescence was measured at 37 °C (λ_{ex} = 560 nm, λ_{em} = 590nm) by a plate reader (Tecan Safire).

For live/dead assay. FDA/PI double-staining were used to label cells for 25 min and washed three times with PBS. Then, the fluorescence images were performed on confocal laser-scanning microscope.

Flow cytometry assay. The counts of MCF-7, HeLa, HepG2, LX-2 cells were treated with the localized circuits. The cells were seeded in a 6-well plate with the density of 2.5 × 10^6 cells/well. After 12 h, the localized circuit was added and incubated for another 12 h. The cells were collected and washed with PBS several times. Subsequently, the counts of cells were measured by the flow cytometer (BD FACSCalibur, FACS101).

qRT-PCR measurement of HIF-1α and EGR-1 expression. The MCF-7 cells were seeded in a 6-well plate with the density of 2.5 × 10^6 cells/well. After 12 hours, the different circuits were added and incubated for co-incubation for 12 hours. The cells were collected and washed with PBS three times. Total RNA was extracted from MCF-7 cells by Trizol Reagent (KGA1203) according to the manufacture's protocol.

Western blot measurement of HIF-1α and EGR-1 protein expression. The MCF-7 cells were seeded in a 6-well plate with the density of 2.5 × 10^6 cells/well. After

incubation for 12 h, the different circuits were added and incubated for 12 hours. The cells were collected and washed with PBS three times. Western blot measurement of HIF-1α and EGR-1 protein expression according to the manufacture's protocol.

Methodology of mice experiment. Female NOD-SCID mice were used for detecting breast cancer xenograft tumour cell progression of MDA-MB-231 cells which were injected subcutaneously. The experiment was approved by the Authority for Biological and Biomedical Models at the Hebrew University, ethical number is NS-21-16745-4. MDA-MB-231 of 9×10^6 cells/ mouse were injected subcutaneously to the flack of each mouse. Tumour mass was generated after 7 days in a volume that is around 80-100 mm^3, then the injections of the treatment were done intra-tumoral (IT) (3 times/week) in total 9 injections, by using circuits (PBS-i, ii, iii, iv, v and vi), all circuits were injected in a volume of 50μl of the amount of 10μM/mouse. For each group we used 4 mice. Tumour was majored every 2-3 days before the following injection to evaluate the width and the height, then tumour volume (mm^3) was measured using the equation of $(Width^2\times Hight)/2$. Toxicity of the treatment was evaluated by the mice weight change (g) that was measured once a week. All results were presented as mean ± SEM.

Experimental method for staining the tumours and the histopathological evaluation of the apoptosis of the different tissue samples. Xenograft tumours of MDA-MB-231 extracted, were fixed in 4% paraformaldehyde, processed for paraffin mounting, and then sectioned, and finally stained using Hematoxylin and eosin (H&E) staining.[a] Then the slides were evaluated to check for abnormal cells, using optical microscopy. High power field (HPF) figures were recorded. For each group we used 3 mice. Each mouse 10 fields were evaluated randomly. All results were measured for the average number of dead cells per HPF, and represented as mean ± SEM. Apoptosis/necrosis of cells was evaluated according to the literature method.[b] Each tumour sample was subjected to analysis of 10 tissue sections.

a. Ben-Hamo, Rotem, et al. "Predicting and affecting response to cancer therapy based on pathway-level biomarkers." *Nat. Commun.* 2020, 11, 3296.

b. Elmore, Susan A., et al. "Recommendations from the INHAND apoptosis/necrosis working group." *Toxicol. Pathol.* 2016, 44, 173-188.

Estimates of entropy and free energy values associated with the entropy-driven evolved [2 × 2] CDN K depicted in Figure 1.

The entire process is shown as a reaction equation in Figure 1B with the corresponding thermodynamic parameters. According to the Gibbs free energy equation:

$$(1)\,\Delta G = \Delta H - T\Delta S$$

The total number of base pairs and the complementary region in the reactants and products are unchanged, giving ΔH ≈ 0. Thus, the reaction is driven forward thermodynamically by the entropic gain of the liberated molecules, and the driving force, at any time, is TΔS. The final concentrations of all species in this entropy-driven DNA circuit can be approximated. According to the van't Hoff equation, the Gibbs free energy change is given by

$$(2)\,\Delta G = 2\Delta G^0_{M_1} + 2\Delta G^0_{M_2} + \frac{1}{2}\Delta G^0_{AA_1} + \frac{1}{2}\Delta G^0_{AB_1} + \frac{1}{2}\Delta G^0_{BA_1} + \frac{1}{2}\Delta G^0_{BB_1} - \Delta G^0_{S_1} - \Delta G^0_{S_2} - \Delta G^0_{A_1}$$
$$-\Delta G^0_{B_1} + RT\ln Q$$

where Q is the reaction quotient, R is the gas constant (8.314 J/mol·K), T is the temperature in K, and ΔG^0_x is the standard free energy of species X under standard conditions that could be obtained by using NUPACK software, giving

$$(3)\,\Delta G = 2\Delta G^0_{M_1} + 2\Delta G^0_{M_2} + \frac{1}{2}\Delta G^0_{AA_1} + \frac{1}{2}\Delta G^0_{AB_1} + \frac{1}{2}\Delta G^0_{BA_1} + \frac{1}{2}\Delta G^0_{BB_1} - \Delta G^0_{S_1} - \Delta G^0_{S_2} - \Delta G^0_{A_1}$$
$$-\Delta G^0_{B_1} = 2.09 \text{ kcal/mol}$$

When the reaction is at equilibrium, which means ΔG = 0, the reaction quotient (Q) could be calculated as 0.033 in our experimental conditions, according to equations 2 and 3.

Meanwhile,

$$Q = \left\{\left([M_1]/c^0\right)^2\left([M_2]/c^0\right)^2\left([AA_1]/c^0\right)^{\frac{1}{2}}\left([AB_1]/c^0\right)^{\frac{1}{2}}\left([BA_1]/c^0\right)^{\frac{1}{2}}\left([BB_1]/c^0\right)^{\frac{1}{2}}\right\}/$$
$$\left\{\left([S_1]/c^0\right)\left([S_2]/c^0\right)\left([A_1]/c^0\right)\left([B_1]/c^0\right)\right\}$$

Providing that the initial concentrations of A_1, B_1, S_1 and S_2 are 1 μM and that the final concentrations of M_1, M_2 is x (expressed in units of μM) and c^0=1 M. Thus, we can write the following equation:

$$\frac{4\left(10^{-6}x\right)^6}{10^{-24}\left(1-x\right)^4} = 0.033$$

Using the bisection method, x can be estimated to be between 0.99 and 0.999 μM, indicating that a potential reaction efficiency of entropy-driven DNA circuit could be more than 99 % without regard to the reaction time.

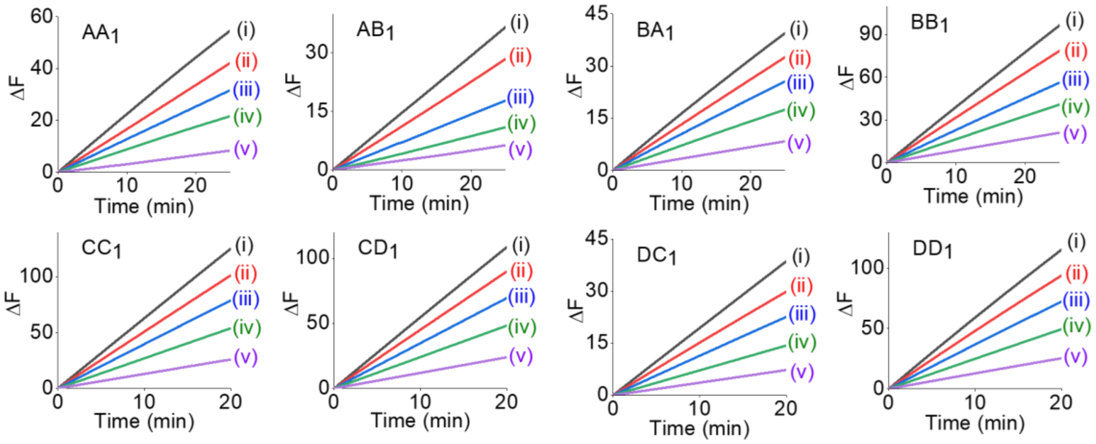

Figure S1. Time-dependent fluorescence changes generated upon the cleavage of the fluorophore/quencher-modified substrates by the respective DNAzyme reporter units associated with the individual intact constituents at variable concentrations: (i) 1 μM, (ii) 0.8 μM, (iii) 0.6 μM, (iv) 0.4 μM, and (v) 0.2 μM.

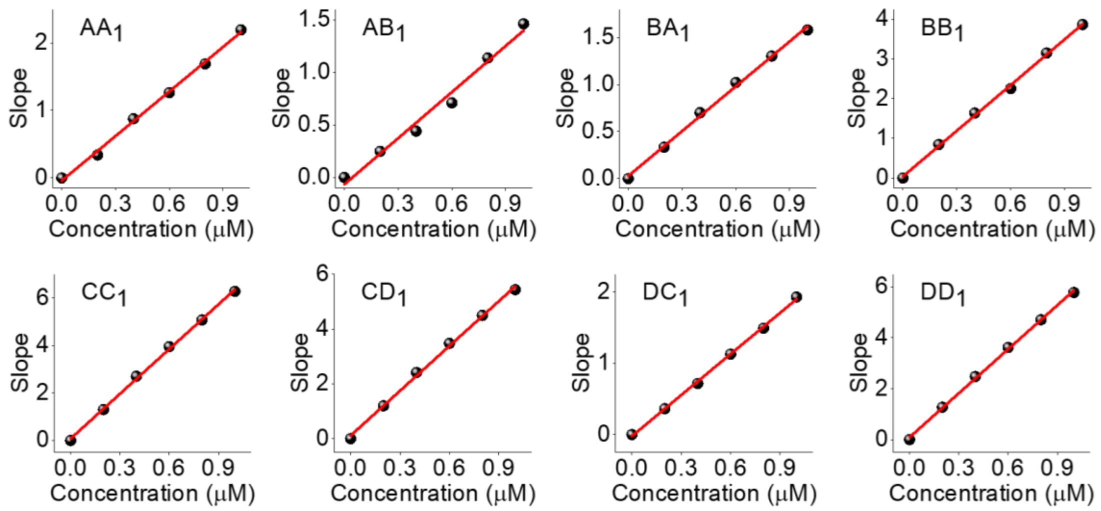

Figure S2. Corresponding calibration curves of the catalytic rates of the different constituents as a function of their concentrations, derived from the data shown in **Figure S1**.

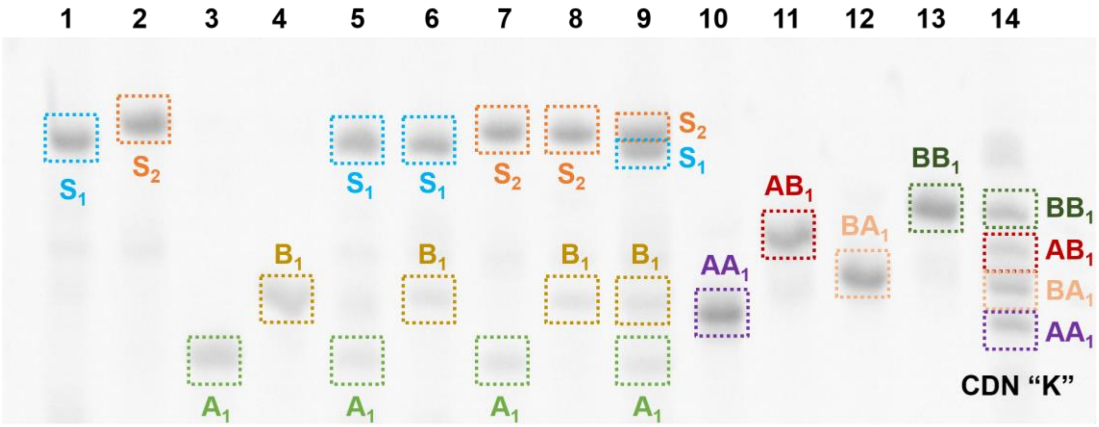

Figure S3. Gel electrophoretic image (15% PAGE) of the separated bands generated, upon the primer-P_1-induced emerged formation of CDN “K” displayed in Figure 1. Lanes 1-4 correspond to the bands of the individual substrate and fuel strands: S_1 (lane 1), S_2 (lane 2), A_1 (lane 3), and B_1 (lane 4). Lanes 5-9 corresponds to the mixture of substrate and fuel strands in the absence of primer P_1: S_1+A_1 (lane 5), S_1+B_1 (lane 6), S_2+A_1 (lane 7), S_2+B_1 (lane 8), and S_1+S_2+A_1+B_1 (lane 9). For comparison, the bands of the separated intact constituents, i.e., AA_1 (lane 10), AB_1 (lane 11), BA_1 (lane 12), and BB_1 (lane 13) are provided. Lane 14 shows the separated constituents of CDN “K”, generated upon P_1-activated entropy-driven DNA circuit shown in Figure 1A.

From the intensities of the stained separated bands for evolved CDN “K” shown in lane 14 and using the stained bands of the individual intact constituents AA_1, AB_1, BA_1 and BB_1 at known concentrations (1 μM), we evaluated, using Image J software, the contents of the constituents in CDN “K”, Table S1. As expected, we find that the contents of the constituents in the electrophoretically separated mixture of CDN “K” is similar to those evaluated by the DNAzyme reporter units, cf. Table 1 in the text.

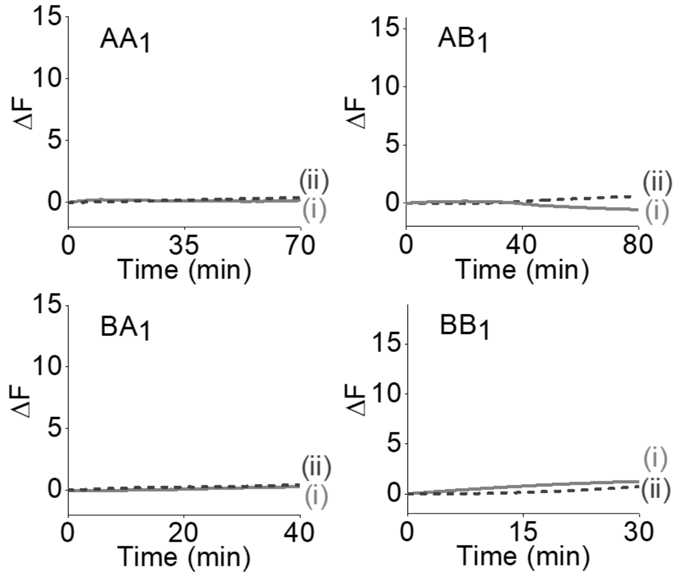

Figure S4. Time-dependent fluorescence changes generated by the Mg^{2+}-ion-dependent DNAzyme reporter units: (i) in the absence of primer P_2, and (ii) upon subjecting P_2 to the separate downstream subcircuit C1.

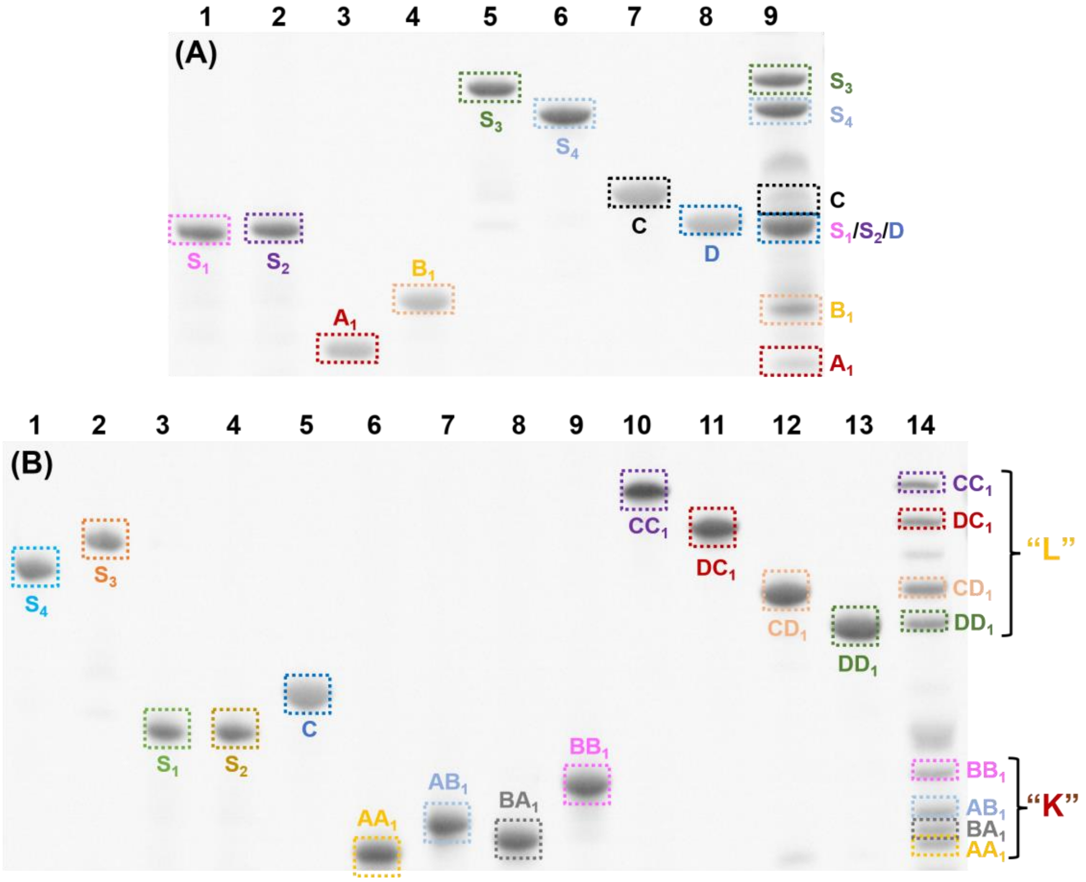

Figure S5. Gel electrophoresis demonstrating the primer-P_2-guided cascaded evolution of CDN "K" and CDN "L", displayed in Figure 2. (A) Electrophoretic bands (15% PAGE) corresponding to the set of substrate and fuel strands in the absence of P_2. Lane 1: S_1, lane 2: S_2, lane 3: A_1, lane 4: B_1, lane 5: S_3, lane 6: S_4, lane 7: C, lane 8: D, lane 9: the mixture of eight substrate and fuel strands in the absence of P_2. (B) Electrophoretic separated bands (15% PAGE) corresponding to the CDN "K" and "L", generated upon the P_2-induced two-layer entropy-driven DNA cascade. Lanes 1-5 corresponding to the individual substrate and fuel strands: lane 1: S_4, lane 2: S_3, lane 3: S_1, lane 4: S_2, lane 5: C. Lanes 6-13 corresponding to the intact separated constituents: lane 6: AA_1, lane 7: AB_1, lane 8: BA_1, lane 9: BB_1, lane 10: CC_1, lane 11: DC_1, lane 12: CD_1, and lane 13: DD_1. Lane 14: the separated bands of evolved CDNs "K" and "L", generated upon the treatment of the mixture of eight substrate and fuel strands with P_2. The results shown in lane 14 demonstrate the P_2-guided cascaded emergence of the CDNs "K" and "L" comprising of the eight constituents AA_1, AB_1, BA_1, BB_1, CC_1, CD_1, DC_1, and DD_1.

Using ImageJ software and comparing the intensities of the separated bands to those of the individual constituents at known concentrations (1 μM), the contents of the constituents in CDNs "K" and "L" generated upon the P_2-induced two-layer entropy-driven evolution cascade were evaluated, and the results were summarized in Table S2.

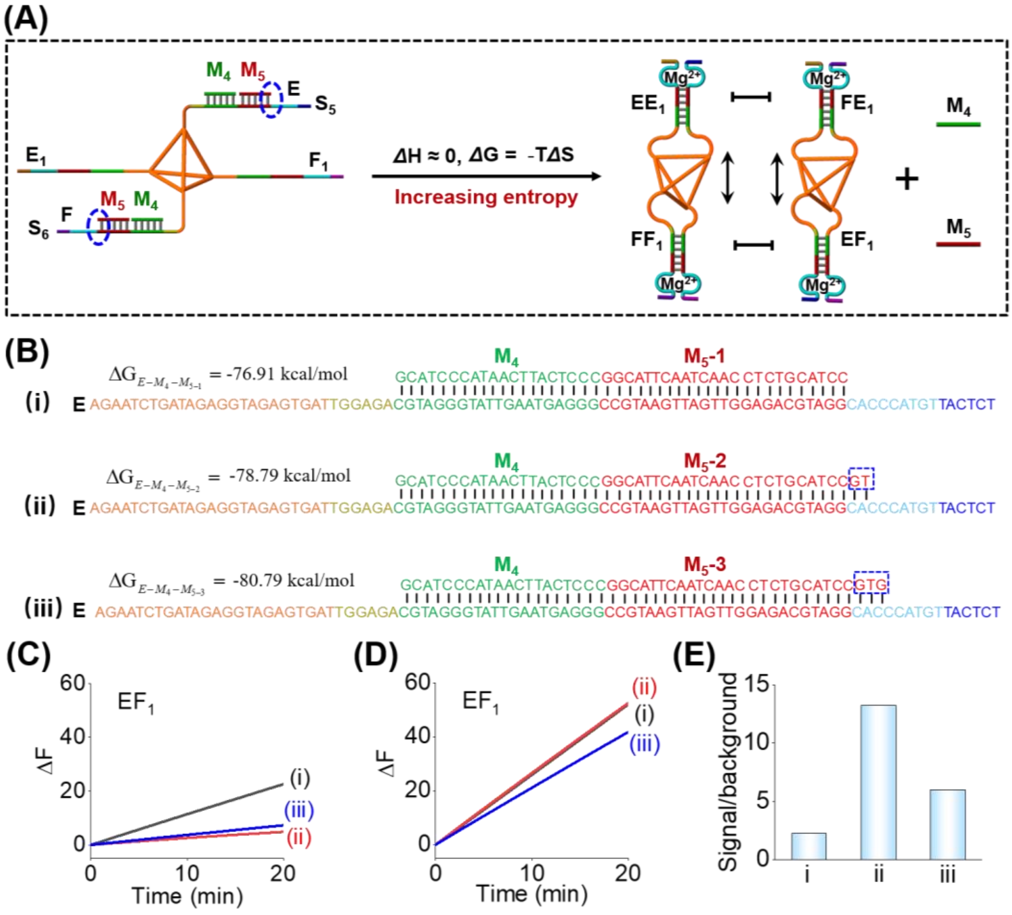

Figure S6. (A) Schematic thermodynamic parameters associated with the P_4-triggered entropy-driven transition of the spatially localized reaction module into CDN "M" and M_4/M_5 products. (B) Detailed sequence information for the optimization of the localized circuit. Time-dependent fluorescence changes generated by the DNAzyme reporter units (C) before subjecting the localized system to P_4 and (D) after P_4-triggered entropy-driven transition of the spatially localized reaction module consisting of: (i) E-M_4-M_{5-1}, (ii) E-M_4-M_{5-2}, and (iii) E-M_4-M_{5-3}.

Optimization of the DNA-tetrahedra reaction circuit composition leading to the primer-induced, entropy-driven, reconfiguration of M_4/M_5-blocking tetrahedra framework shown in Figure 3A.

We note that the primer-induced entropy-driven localized reconfiguration of the DNA-tetrahedra circuit T_1 to CDN "M" reveals low leakage phenomenon, often associated with strand displacement processes, resulting in high signal-to-noise readout signal. This originates from optimized pre-engineering of the reaction circuits.

The tether E is blocked by the sequences M_4 and M_5. The tethers E_1 and F_1 include sequence domains that could, in principle, lead to the displacement of M_5 thereby activating the reaction circuit in the absence of the primer and resulting in leakage

output signals. Accordingly, the strand M_5 was structurally modified to minimize the leakage phenomenon upon operating the circuit shown in Figure S6A. Three modified M_5 strands, M_{5-1}, M_{5-2}, and M_{5-3}, Figure S6B, were used to operate the circuit in the absence, Figure S6C, and presence, Figure S6D of the primer. (Note that M_{5-2} and M_{5-3} include two and three additional bases, respectively). The results demonstrate that strand M_{5-1} reveals high leakage, whereas strands M_{5-2} and M_{5-3} show low leakage (in the absence of the primer). In turn, in the presence of the primer, strands M_{5-1} and M_{5-2} demonstrate high output signals, whereas strand M_{5-3} shows a lower output signal. The signal-to-noise values of the reaction circuit in the presence of M_{5-1}, M_{5-2}, and M_{5-3}, are summarized in Figure S6E, demonstrating that strand M_{5-2} represents the optimized strand for the circuit (lowest signal-to-noise ratio).

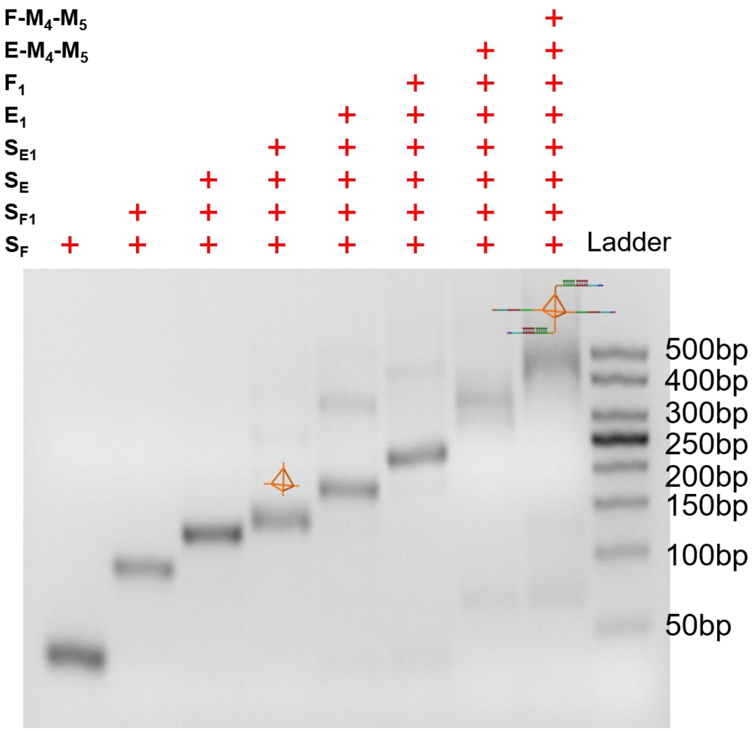

Figure S7. Agarose gel (4%) electrophoretic image demonstrating the spatial organization of circuit elements on tetrahedra T_1.

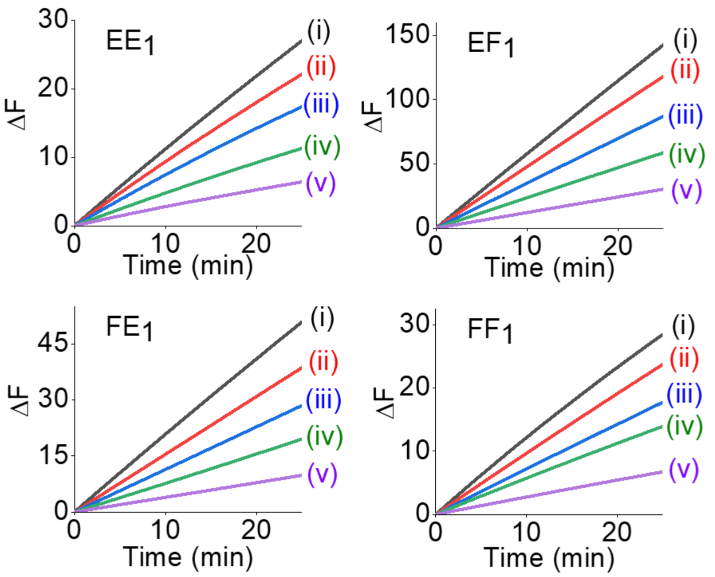

Figure S8. Time-dependent fluorescence changes generated upon the cleavage of the fluorophore/quencher-modified substrates by the respective Mg^{2+}-dependent DNAzyme reporter units associated with the individual intact constituents at variable concentrations: (i) 1 μM, (ii) 0.8 μM, (iii) 0.6 μM, (iv) 0.4 μM, and (v) 0.2 μM.

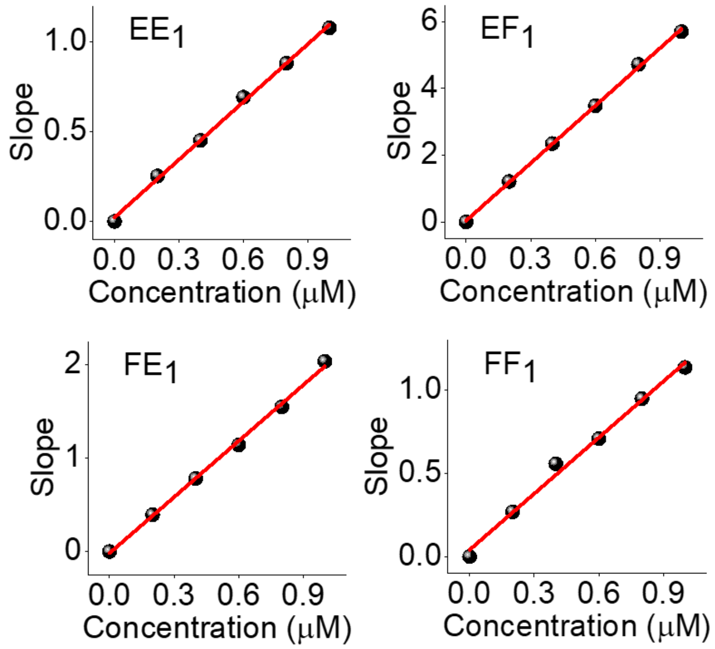

Figure S9. Corresponding calibration curves of the catalytic rates of the different constituents as a function of their concentrations, derived from the data shown in **Figure S8**.

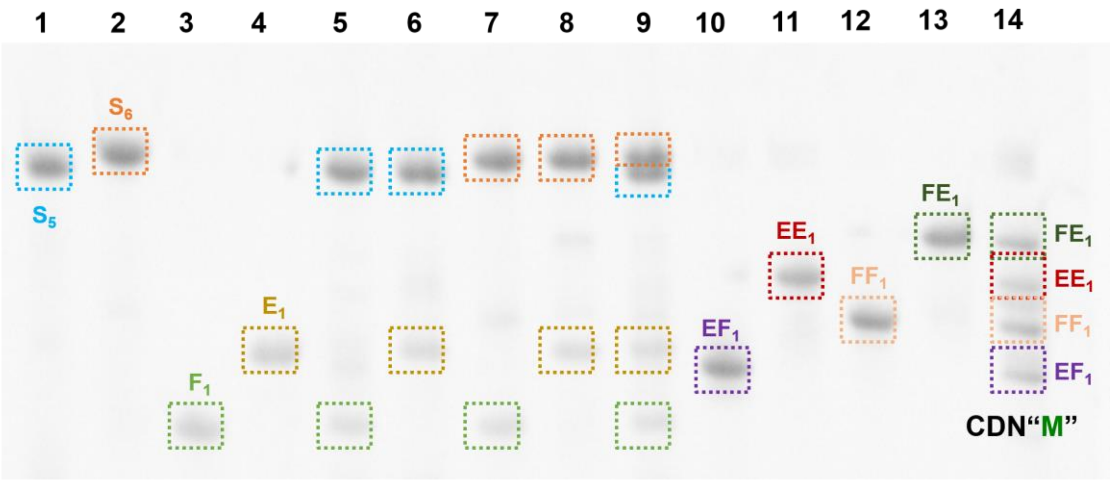

Figure S10. Gel electrophoresis (15% PAGE) demonstrating primer-P_4-guided evolution of CDN "M" displayed in Figure 3. For comparison, the bands of the individual substrate and fuel strands, namely, S_5 (lane 1), S_6 (lane 2), F_1 (lane 3), and E_1 (lane 4), the mixture of substrate and fuel strands in the absence of primer P_4: S_5+F_1 (lane 5), S_5+E_1 (lane 6), S_6+F_1 (lane 7), S_6+E_1 (lane 8), and S_5+S_6+E_1+F_1 (lane 9), and the separated intact constituents, EF_1 (lane 10), EE_1 (lane 11), FF_1 (lane 12), and FE_1 (lane 13) are provided. Lane 14 shows the separated constituents of CDN "M", generated upon the P_4-triggered activation of entropy-driven DNA circuit.

Using ImageJ software and comparing the intensities of the separated bands to those of the individual constituents at known concentrations (1 μM), the contents of the constituents in CDNs "M" were evaluated, and the results were summarized in Table S3.

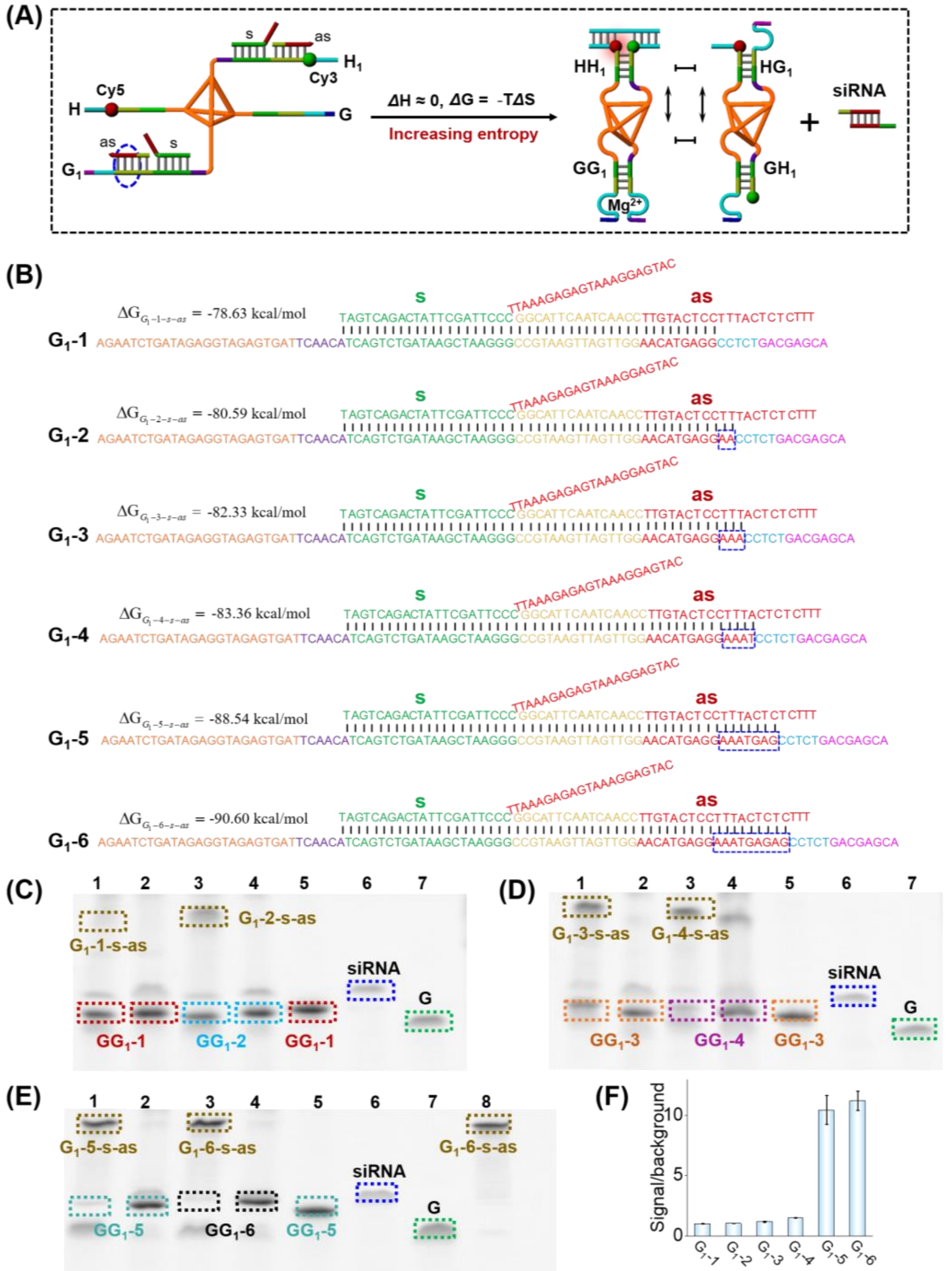

Figure S11. (A) Schematic thermodynamic parameters associated with the miR-21-triggered spatially localized reaction module shown in Figure 4A. (B) Detailed sequence information for the optimization of the proposed localized circuit. (C) Gel electrophoresis analysis of miR-21-triggered entropy-driven transition of the spatially localized reaction module consisting of: G_1-1-s-as or G_1-2-s-as. (D) Gel electrophoresis analysis of miR-21-triggered entropy-driven transition of the spatially localized reaction module consisting of: G_1-3-s-as or G_1-4-s-as. (E) Gel electrophoresis analysis of miR-21-triggered entropy-driven transition of the spatially localized reaction module consisting of: G_1-5-s-as or G_1-6-s-as. (F) Quantitative evaluation of the ratio of signal and background shown in Figure S11C-11E.

Optimization of the DNA-tetrahedra reaction circuit composition leading to the primer-induced, entropy-driven, reconfiguration of s/as-blocking tetrahedra framework shown in Figure 4A.

We note that the primer-induced entropy-driven localized reconfiguration of the DNA-tetrahedra circuit T_2 to CDN "N" reveals low leakage phenomenon, often associated with strand displacement processes, resulting in high signal-to-noise readout signal. This originates from optimized pre-engineering of the reaction circuits. The tether E is blocked by the sequences s and as. The tethers E_1 and F_1 include sequence domains that could, in principle, lead to the displacement of s/as thereby activating the reaction circuit in the absence of the primer and leading to leakage output signals. Accordingly, the strand G_1 was structurally modified to minimize the leakage phenomenon upon operating the circuit shown in Figure S11A. Six modified G_1 strands, G_1-1, G_1-2, G_1-3, G_1-4, G_1-5, and G_1-6, Figure S11B, were used to operate the circuit in the absence and presence of the primer, Figure S11C-E. (Note that strands G_1-1 - G_1-6 include 2, 3, 4, 7, 9 additional bases). The results demonstrate that G_1-1 - G_1-4 reveal high leakage, whereas strand G_1-5 and G_1-6 show low leakage (in the absence of the primer). In turn, in the presence of the primer, all strands G_1-5 - G_1-6 demonstrate high output signals. The signal-to-noise values of the reaction circuit in the presence of strands G_1-1 - G_1-6 are summarized in Figure S11F demonstrating that strand G_1-6 represents the optimized strand for the circuit (lowest signal-to-noise ratio).

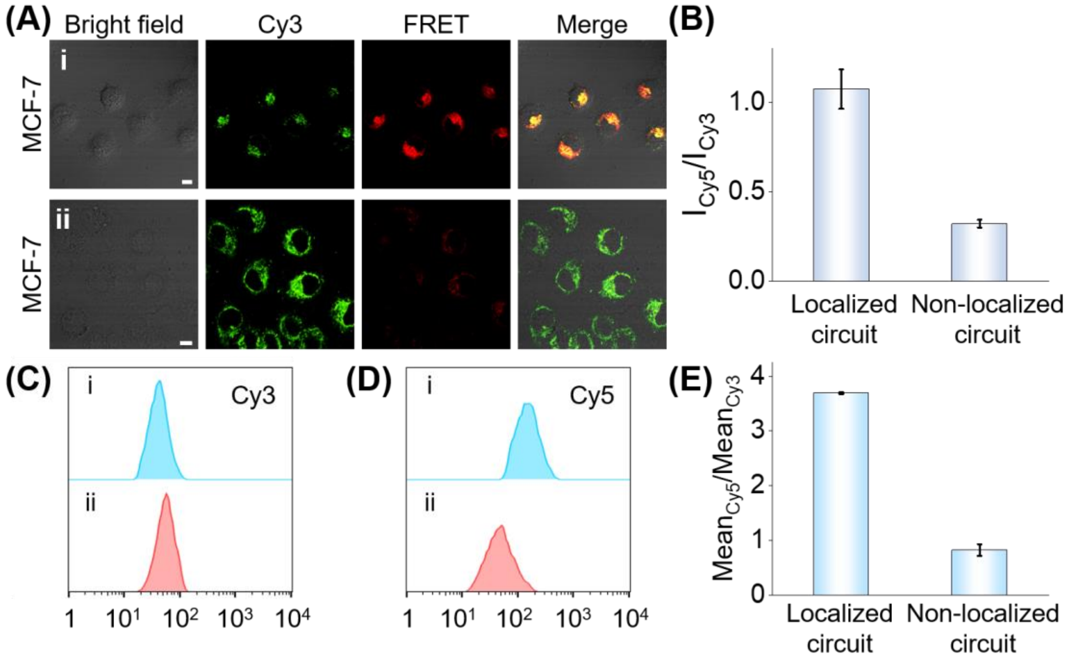

Figure S12. (A) Confocal fluorescence imaging of MCF-7 cells treated with localized entropy-driven DNA circuits (i) and non-localized entropy-driven DNA circuits (ii). (B) Statistical histogram analysis of the fluorescence intensity ratio (I_{Cy5}/I_{Cy3}) derived from the respective confocal microscopy images. Flow cytometry analysis of Cy3 fluorescence (C) and Cy5 fluorescence (D) of MCF-7 cells treated with localized entropy-driven DNA circuits (i) and non-localized entropy-driven DNA circuits (ii). (E) Quantitative flow cytometry analysis of fluorescence intensity ratio (I_{Cy5}/I_{Cy3}) of the above samples.

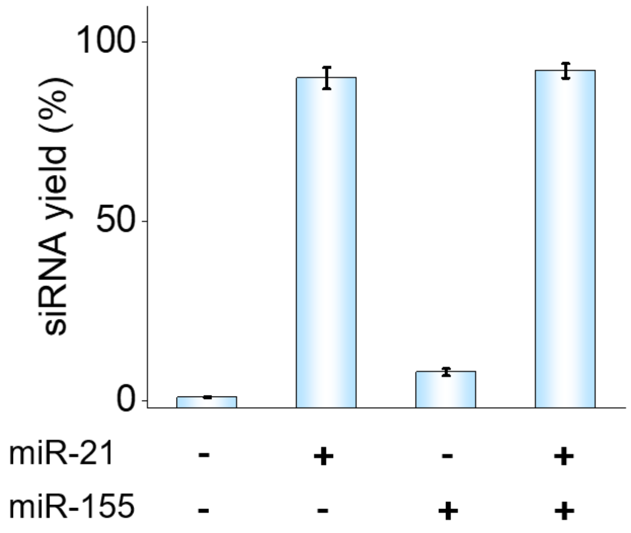

Figure S13. The corresponding quantification analysis of gel electrophoresis shown in Figure 6A.

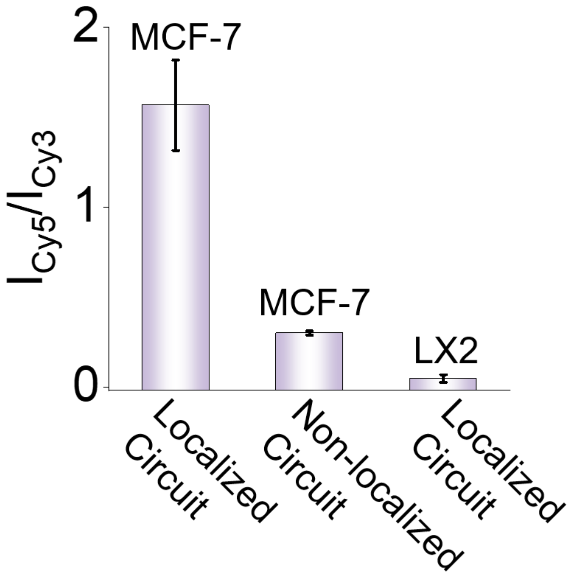

Figure S14. Statistical histogram analysis of the fluorescence intensity ratio (I_{Cy5}/I_{Cy3}) derived from confocal microscopy images in Figure 6D.

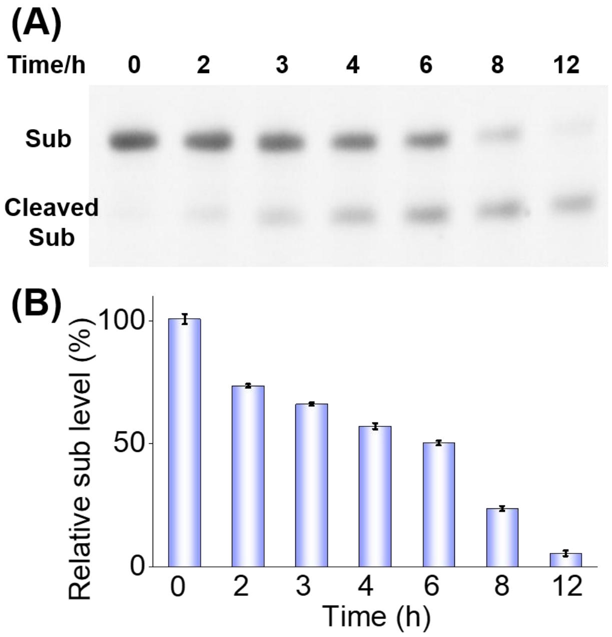

Figure S15. (A) Gel electrophoresis analysis of cleavage efficiency of the EGR-1 mRNA by self-assembled DNAzyme associated with the constituent GG_1 at different time interval, and (B) the corresponding quantification analysis of gel electrophoresis.

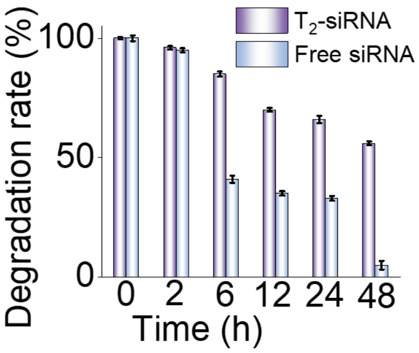

Figure S16. Analysis of stability of siRNA-functionalized tetrahedra T_2 and free siRNA in culture medium containing 10 % fetal bovine serum.

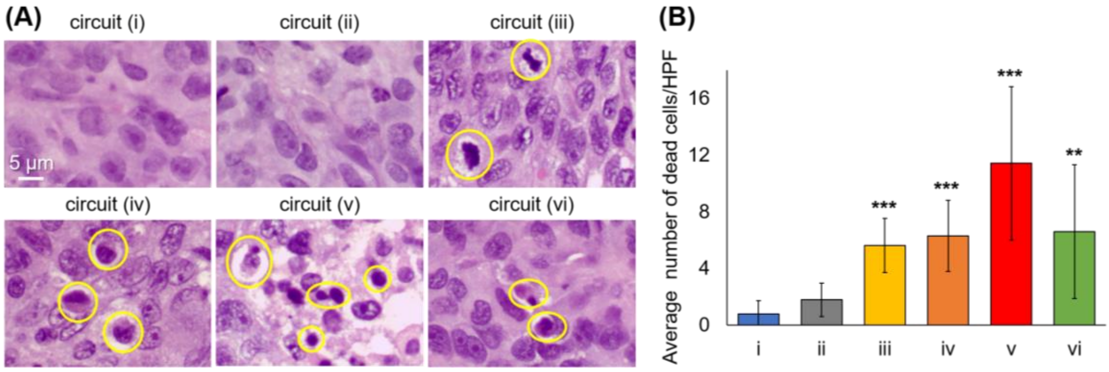

Figure S17. Histopathological evaluation of the average number of apoptotic cells per HPF, corresponding to MDA-MB-231 xenograft tumours treated with PBS (i), inert tetrahedron (ii), circuitries (iii-vi), after staining tumours with H&E. (A) HPF figures representing the stained sections of the different samples after staining them with H&E, yellow circles show the abnormal cells corresponding dead cells. (B) Column graph presenting the average number of dead cells per HPF in the different treatments. All results were presented as mean ± SEM. Significant results were evaluated using T-test; *$**P<0.01$, $***P<0.001$*.

Histopathological evaluation of the tumors treated with the different gene therapeutic circuits

Figure S17A exemplifies the histopathological images of the MDA-MB-231 tumors treated for 28 days with the gene-therapeutic circuits (iii)-(vi) and control systems consisting of pure buffer (i) and inert DNA tetrahedra (ii). The staining procedure and analysis of the apoptotic cells are detailed in the experiment section. The apoptotic (dead) cells for each of the samples are marked with yellow circles. By analyzing ten different tumors slices of each of the tissue samples, the average number of apoptotic cells in the respective samples were estimated, and the results are summarized in Figure S17B. The histopathological experiments following the degree of apoptotic (dead) cells further confirm the cell experiments and *in vivo* IT-treated tumor growth inhibition studies by the different circuits.

Table S1. The contents of the constituents in CDN "K" evolved by the primer P_1-triggered activation of entropy-driven DNA circuit shown in Figure 1.

CDN	AA_1	AB_1	BA_1	BB_1
K[a]	0.43	0.34	0.40	0.46

[a] The contents of the constituents (μM) were evaluated from the quantitative electrophoretic experiments in Figure S3.

Table S2. The composition of the constituents in CDN "K" and CDN "L" evolved by the primer P_2-triggered activation of the two-layer cascaded entropy-driven DNA circuit, shown in Figure 2.

**CDN**	**AA_1**	**AB_1**	**BA_1**	**BB_1**	**CDN**	**CC_1**	**CD_1**	**DC_1**	**DD_1**
**K**[a]	0.13	0.17	0.20	0.15	**L**[a]	0.40	0.40	0.38	0.47
**K**[b]	0.17	0.20	0.23	0.16	**L**[b]	0.36	0.32	0.45	0.40

[a] The contents of the constituents (μM) were evaluated from the time-dependent fluorescence changes generated by the reporter units and using appropriate calibration curves in Figures S1 and S2.

[b] The contents of the constituents (μM) were evaluated from the quantitative electrophoretic experiments in Figure S5.

Table S3. The composition of the constituents in CDN "M" evolved by the primer P_4-triggered activation of spatially localized DNA circuit shown in Figure 3.

**CDN**	**EE_1**	**EF_1**	**FE_1**	**FF_1**
M[a]	0.46	0.46	0.42	0.40
M[b]	0.40	0.35	0.46	0.50

[a] The contents of the constituents (μM) were evaluated from the time-dependent fluorescence changes generated by the reporter units and using appropriate calibration curves in Figures S8 and S9.

[b] The contents of the constituents (μM) were evaluated from the quantitative electrophoretic experiments in Figure S10.
